# Mechanistic insights of ABC importer HutCD involved in heme internalization by *Vibrio cholerae*

**DOI:** 10.1038/s41598-022-11213-9

**Published:** 2022-05-03

**Authors:** Indrila Saha, Shrestha Chakraborty, Shubhangi Agarwal, Peeali Mukherjee, Biplab Ghosh, Jhimli Dasgupta

**Affiliations:** 1 Department of Biotechnology, St. Xavier’s College (Autonomous), 30, Mother Teresa Sarani, Kolkata, 700016 India; 2 Macromolecular Crystallography Section, Beamline Development & Application Section, Bhabha Atomic Research Center, Trombay, Mumbai 400085 India; 3 Present Address: Weill Cornell Medicine, Department of Anesthesiology, 1300 York Ave, New York, NY 10065 USA

**Keywords:** Computational biophysics, Membrane biophysics, Membrane structure and assembly, Enzymes

## Abstract

Heme internalization by pathogenic bacteria inside a human host to accomplish the requirement of iron for important cellular processes is of paramount importance. Despite this, the mechanism of heme import by the ATP-binding-cassette (ABC) transporter HutCD in *Vibrio cholerae* remains unexplored. We have performed biochemical studies on ATPase HutD and its mutants, along with molecular modelling, docking and unbiased all-atom MD simulations on lipid-solvated models of permease-ATPase complex HutCD. The results demonstrated mechanisms of ATP binding/hydrolysis and trapped transient and global conformational changes in HutCD, necessary for heme internalization. ATPase HutD forms a dimer, independent of the permease HutC. Each HutD monomer canonically binds ATP in a 1:1 stoichiometry. MD simulations demonstrated that a rotational motion of HutC dimer occurs synchronously with the inter-dimeric D-loop interactions of HutDs. F151 of TM4–TM5 loop of HutC, packs with ATP and Y15 of HutD, initiating ‘cytoplasmic gate opening’ which mimics an ‘outward-facing’ to ‘inward-facing’ conformational switching upon ATP hydrolysis. The simulation on ‘inward-facing’ HutCD culminates to an ‘occluded’ state. The simulation on heme-docked HutCD indicated that the event of heme release occurs in ATP-free ‘inward-facing’ state. Gradual conformational changes of the TM5 helices of HutC towards the ‘occluded’ state facilitate ejection of heme.

## Introduction

Motile pathogenic bacteria acquire iron, the essential metal cofactor for several cellular processes, aiming at successful colonization and growth inside a host^1–3^. Animal hosts attempt to limit iron availability through ‘nutritional immunity’ thereby combating the colonization of pathogens^4^. This results in free-iron levels as low as 10^−24^ M^5^. To refute this, many pathogens develop various efficient strategies to obtain iron from the host-derived molecules during infection. The main strategies for iron acquisition by pathogenic bacteria include direct extraction of Fe^3+^ from specific iron-containing complexes of the hosts, such as lactoferrin, transferrin, haemoglobin or hemin, and/or production of small Fe^3+^-chelating molecules called siderophores^6,7^. Heme or hemin is considered beneficial for pathogens due to its abundance within the host environment, especially when the pathogen lacks the ability to synthesize heme, as observed in the case of some heme auxotrophs^8^. The mechanisms of ‘iron thievery’ of pathogenic bacteria require special attention since these can be mimicked for the ‘Trojan Horse’ mechanism of antibiotic delivery to diminish permeability-mediated drug resistance^9^.

The structure of the cellular envelope of gram-negative bacteria requires heme to be trafficked across the outer membrane, through the periplasm, and across the inner membrane before reaching the cytosol. Import of heme across the outer membrane of bacteria is accomplished by a TonB-dependent system with the help of proton motive force^10^. Active transport of the nutrients across the plasma membrane is achieved by various ATP binding cassette (ABC) transporters. Generally, periplasmic ligand binding protein (PLBP) captures nutrient, cytosolic nucleotide binding domains (NBDs) utilise ATP hydrolysis driven energy to cause large-scale conformational changes in the permeases to facilitate uptake of nutrients. Interestingly, despite their common basic architecture, molecular mechanisms of ATP binding and hydrolysis mediated alteration of nutrient translocation pathway are remarkably diverse even for the Type II importers, which are well known for the uptake of iron-siderophores, vitamin B12 and heme^11–15^. So far, structural details of heme transporting ABC importers are restricted to HmuUV of *Yersinia pestis* and BhuUV-T of *Burkholderia cenocepacia*^13,14^. Crystal structure of HmuUV of *Y. pestis* demonstrated an ‘outward-facing’ (OF) conformation of the permeases with the translocation pathway opening to the periplasmic side of the membrane. The structure of BhuUV-T of *B. cenocepacia*, on the other hand, was obtained in ‘inward-facing’ (IF) conformation with the substrate translocation pathway open to the cytoplasm^14^. Recently, a targeted MD simulations on BhuUV-T with bound ATP in the IF state yielded an ‘occluded’ (Occ) state, in which both the cytoplasmic and periplasmic sides of heme translocation channel remained closed^16,17^.

*Vibrio cholerae*, the causative agent of diarrheal disease cholera, encodes multiple iron acquisition systems, including the synthesis and transport of the catechol-type siderophore vibriobactin, transport of exogenous siderophores such as enterobactin and the acquisition of heme of the host system^18,19^. Analysis of the hut genes indicated that HutCD-B, which encodes a periplasmic binding protein (HutB), a cytoplasmic membrane permease (HutC) and an ATPase (HutD) are required to reconstitute the *V. cholerae* heme-transport system in *Escherichia coli*^20^. Based on that, ABC type transport system HutCD-B has been identified as the system of *V. cholerae* for heme transportation through the inner membrane^21^. However, the structure, dynamics and mechanism of ATP-dependent heme translocation through this system of *V. cholerae* remains unknown. As an early step to understand the molecular basis of HutCD-B mediated heme transport, we solved the crystal structure of periplasmic heme-binding protein HutB^22^. Structural studies coupled with biochemical analysis indicated parallel binding of two heme molecules to HutB in a pH-dependent manner which attributed to its storage^22^. MD simulation studies exhibited an unforeseen inter-lobe swinging motion that is presumably important for binding and/or ejection of heme to the permeases. Nonetheless, the mechanism of heme internalization is still unknown in *V. cholerae*.

In ABC transporters, the nature of conformational changes is largely specific to the species and the nutrients. Functional assays with HutD and its mutants along with molecular modelling, docking and MD simulations on HutCD assemblies were performed here to identify the pivotal residues involved in ATP binding/hydrolysis, as well as to trap the ATP hydrolysis driven transient and global conformational changes required for heme internalization. Here we have prepared structural models of HutCD in the OF and IF states. Two Mg^2+^-ATP molecules were docked in HutDs of OF states. HutCD in the IF state was docked with heme. The HutCD assemblies in both the states were inserted into a solvated lipid (dimyristoyl phosphatidylcholine, DMPC) bilayer. To get insights into the conformational shifts expected to occur in the heme transportation pathway in ± Mg^2+^-ATP states, unbiased MD simulations were performed on all the sets for 1 μs each. Biochemical results endorsed that the NBD, HutD forms dimer and like other type-II importers, each monomer binds one molecule of ATP. MD simulations on membrane embedded assemblies of HutCD delineated the intricate details required to execute coupled rotational motions between HutCs and HutDs in the IF and OF states. Probable pathway and interactions of heme with HutCD during ejection from the ‘cytoplasmic gate’ to the cytoplasm have been identified through MD simulations on the heme docked HutCD complex.

## Results

### Sequence analysis of HutC and HutD

**Figure 1:**
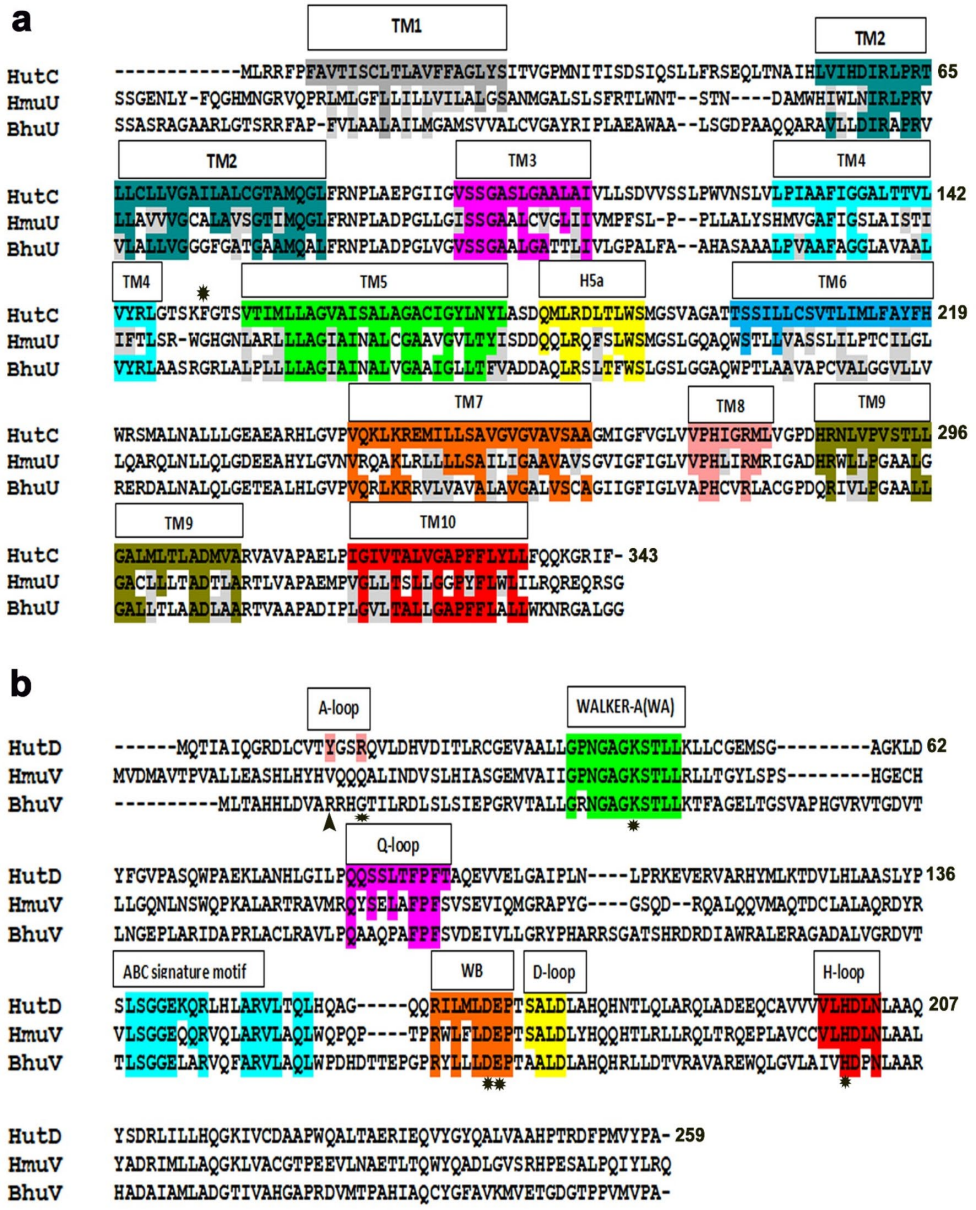
Sequence analysis of HutC and HutD. (**a**) Sequence alignment of HutC from *Vibrio cholerae*, HmuU from *Yersinia pestis* and BhuU from *Burkholderia cenocepacia*. Numbering is based on HutC sequence. (**b**) Sequence alignment of HutD from *Vibrio cholerae*, HmuV from *Yersinia pestis* and BhuV from *Burkholderia cenocepacia*. Numbering is based on HutD sequence. Important motifs and conserved residues are individually coloured/marked. Semi-conservation inside important motifs are shaded in grey. Some important residues are marked with ‘*’.

To structurally characterize HutCD of *V. cholerae* we have performed a BLAST search against PDB with the amino acid sequences of the TMD, HutC (Accession code: C3LWH8) and the NBD, HutD (Accession code: A0A0H3ADP8) and aligned the sequences with their close structural homologs using Clustal Omega (Fig. 1). HutC has shown 45% identity and 67% similarity (for 303aa) with heme transporting permease HmuU of *Y. pestis* and 45% identity (63% similarity for 339 aa) with BhuU, of *B. cenocepacia* (Fig. 1a). In addition to the heme transporters, HutC sequence also showed 41% identity and 58% similarity (for 293 aa) with BtuC, the ABC transporter permease involved in vitamin B12 uptake. HutD, the cytosolic ATPase showed a maximum identity of 45% (62% similarity for 242 aa) with HmuV while identity with BhuV of *B. cenocepacia* was relatively less (36% identity and 51% similarity for 276 aa). Identity of HutD with BtuD of BtuCD was 36% (52% similarity for 217 aa). The significant extent of identity with the heme and B12 ABC transporters of different prokaryotic systems strongly indicates that HutCD is expected to possess a similar overall fold and belongs to the type II class of ABC transporter. The predicted topology of HutC with ten transmembrane helices appears to correspond to the transmembrane segment of the permeases of the type II class (marked in Fig. 1a). Similarly, HutD, like the other NBDs, exhibits sequence and structure conservation across the transporter family^23^ and contains several conserved functional motifs such as Walker-A (WA), Walker-B (WB) motifs, an ABC transporter-specific or ‘signature’ motif, and two shorter sequences containing conserved glutamine and histidine residues (Q-loop and H-loop, respectively) (Fig. 1b).

### Cross-linking and size exclusion chromatography suggest dimerization of HutD

**Figure 2:**
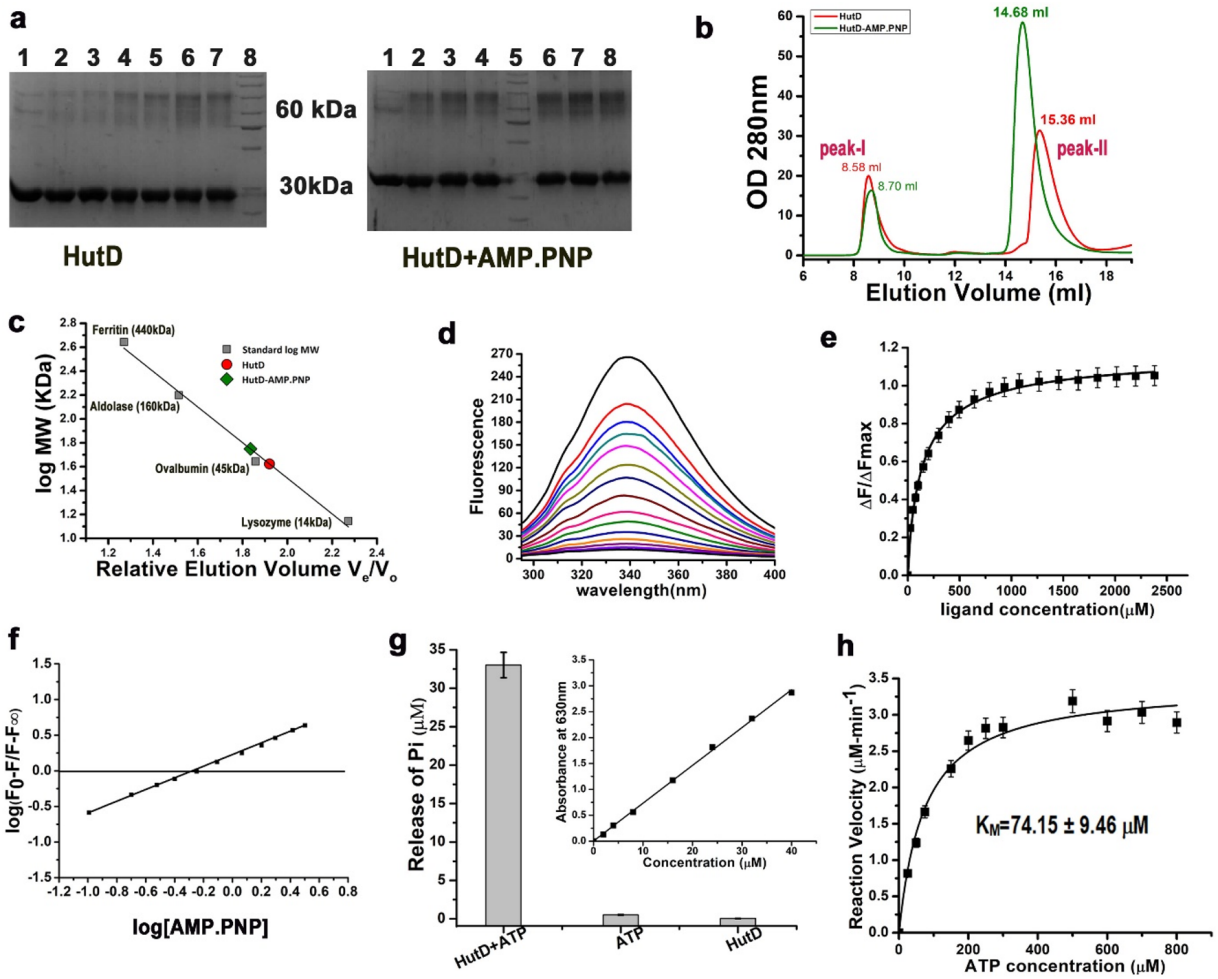
Dimerization and ATP binding/hydrolysis by HutD. (**a**) Crosslinking of HutD with Glutaraldehyde in Nt-free state (left) and after incubation with AMP.PNP showed prevalent dimerization upon incubation with AMP.PNP; *left:* (L1: Control, L2–L7: after treatment with 0.005–0.04% of Glutaraldehyde, L8: MWM), right: (L1: Control, L2–L4: after treatment with 0.005%, 0.01%, and 0.02% of Glutaraldehyde, L5: MWM, L6–L8: after treatment with 0.03%, 0.035%, and 0.04% of Glutaraldehyde). A cropped version is used here to reduce space and to maintain clarity. Original gel pictures are available in Fig. S1; (**b**) size exclusion chromatography elution profiles of HutD in Nt-free and upon incubation with AMP.PNP; (**c**) the molecular weights of the peaks were determined from the calibration curve prepared using molecular weight standards. Elution volumes suggest strong dimeric shift upon AMP.PNP binding to HutD. (**d,e**) Fluorescence quenching demonstrated significant change in fluorescence upon AMP.PNP binding to HutD; (λ_exc_ = 280 nm, λ_em_ = 295–400 nm) with slit widths of 5 nm for both excitation and emission. Plots of ΔF/ ΔF_max_ against ligand AMP.PNP (mM) obtained a K_d_ value of 94 ± 0.024 μM; (**f**) slope of the *straight line* indicates 1:1 binding stoichiometry between HutD and AMP.PNP; (**g**) significant ATP hydrolysis of HutD were observed in Malachite green assay and the release of inorganic phosphate (*Pi*) was estimated against the standard curve of KH_2_PO_4_ (*Inset*); (**h**) reaction velocity of HutD, measured at a protein concentration of 2.5 μM, were plotted against ATP concentrations (μM) as per Michaelis–Menten equation y = Vmax × x^n^/(k^n^ + x^n^) with ‘One site-specific binding’ model. Error bars in (**e,g,h**) are SD values obtained from at least three replicates.

To identify the oligomeric state of HutD, crosslinking experiments were carried out with HutD in the presence and absence of AMP.PNP, the non-hydrolysable analogue of ATP, using Glutaraldehyde as crosslinker (Fig. 2a, Fig. S1). Migration pattern, analysed by 10% SDS-PAGE gel, depicted the presence of dimer (~ 60 kDa) along with monomer of HutD (30 kDa) both in the presence and absence of AMP.PNP (Fig. 2a, Fig. S1). However, tendency of forming dimer is prevalent in the presence of AMP.PNP (Fig. 2a). Little bit of anomaly in movement of proteins in SDS-PAGE (which is observed in case of HutD dimer) is not very uncommon upon cross-linking or chemical modifications of proteins. Moreover. HutD, that was used for cross-linking experiments was pure and functional, and hence the band near ~ 60 kDa has been considered as dimeric band.

To further validate the results of cross-linking experiments, we performed size exclusion chromatography (SEC) using Superdex 200 increase column 10/300 with HutD in free and AMP.PNP treated states (Fig. 2b,c; free state and AMP.PNP treated HutD are shown in red and green colours respectively). In free state, peak II of Fig. 2b having elution volume of 15.36 ml was indicative of monomer along with a trace amount of dimer. However, incubation with AMP.PNP showed a clear shift of peak-II towards dimer with elution volume of 14.68 ml (Fig. 2b,c). This indicates that AMP.PNP binding facilitates dimerization which corroborates with the observations of cross-linking experiments (Fig. 2a). In both experiments of SEC, peak-I probably denotes little impurities in the samples and/or soluble aggerates.

So far, HutCD was known as a putative type-II ABC importer of heme, based on sequence comparisons. Our observations authenticated that like other type-II importers HutD is capable of forming dimer in the absence of the permease, HutC, and ATP binding facilitates dimerization of HutD.

### Binding of AMP.PNP with HutD

Participation of A-loop, Walker-A and Walker-B is well known in the ATP binding in the NBDs of type-II ABC importers (PDB codes: 4G1U, 1L2T). Sequence alignment (Fig. 1b) indicated proximity of Y15 of A-loop to the ATP binding site (Fig. 1b). We, therefore, decided to investigate the interactions of AMP.PNP, the non-hydrolysable ATP analog, with HutD by monitoring the intrinsic fluorescence quenching at an excitation wavelength of 280 nm (λ_exc_ = 280 nm, λ_em_ = 295–400 nm) accounting the contributions of tryptophans and tyrosines present at the ligand binding site. Interestingly, with significant fluorescence quenching, HutD has shown considerable interactions with AMP.PNP with a dissociation constant, K_d_ of 94 ± 0.024 μM (Fig. 2d,e). Calculation of stoichiometry indicated binding between HutD and AMP.PNP in a 1:1 molar ratio (Fig. 2f). Negligible quenching upon excitation at wavelength of 295 nm (λ_exc_ = 295 nm, λ_em_ = 308–400 nm), that accounts the contribution of Trp only, ruled out the participation of the Trp residues in ATP binding (Fig. S2) and established that the Trp residues are located beyond the foster distance of ATP binding site of HutD. In turn, these results established the proximity and participation of Y15 in ATP binding.

### ATP hydrolysis by HutD and calculations of V_max_ and K_M_

HutD has shown substantial ATP hydrolysis while tested using Malachite green assay (Fig. 2g). 2.5 µM HutD and 100 μM ATP (Sigma Aldrich) were incubated at 298 K for 20 min (Fig. 2g). The release of inorganic phosphate (Pi) was measured at 630 nm upon incubation with Malachite green as per the protocol described earlier^24^. Released Pi from each reaction was quantified by comparing with a Pi standard curve prepared using KH_2_PO_4_ (inset of Fig. 2g). HutD without ATP addition served as the control reaction. The experiments were minimally performed in triplicates.

We have determined maximal velocity (V_max_) and Michaelis constant (K_M_) through time course ATPase assays of HutD. Considering linearity of Pi production during time course experiment, reaction velocity was measured upon incubation of HutD with increasing ATP concentrations (up to 800 μM) through a time scan up to 5 min. Reaction velocity (V_0_) in terms of Pi release was then plotted against ATP concentrations (Fig. 2h). Fitting of ATPase activities in Michaelis–Menten equation resulted in V_max_ of 3.42 ± 0.10 μM/min and K_M_ of 74.15 ± 9.46 μM for HutD (Fig. 2h).

### Identification of pivotal functional residues of HutD through mutagenesis

**Figure 3:**
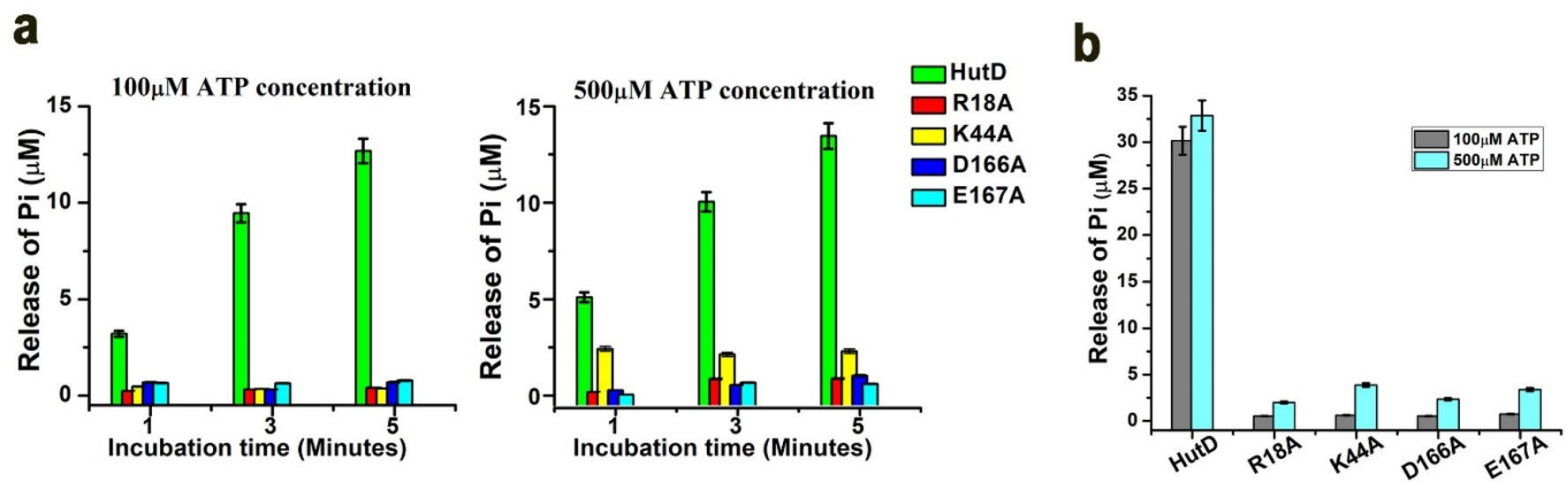
Identification of functionally crucial residues of HutD by mutagenesis. (**a**) Loss of function of the mutants R18A, K44A, D166A, E167A compared to HutD, as evident from the measurement of ATPase activities with 100 μM and 500 µM ATP at three time points (1, 3, 5 min). (**b**) ATPase activities of HutD and mutants measured with an extended incubation time of 20 min with 100 μM and 500 μM of ATP.

Based on sequence similarity, we could presume functionally important residues of HutD (Fig. 1b, Table S1). To confirm our propositions, we have prepared four point mutants of HutD namely R18A, K44A, D166A and E167A, and investigated their ATP hydrolysis capacity using Malachite green assays. Time course experiments showed that the amount of Pi produced by wild type (wt) HutD during ATP hydrolysis increased approximately linearly with incubation time for the first 5 min (Fig. 3a). However, the ATPase activity using 100 μM of ATP of the mutants R18A, K44A, D166A and E167A were drastically low compared to wt HutD and remained constant throughout for different incubation times varying from 1 to 5 min (Fig. 3a). The concentration of ATP inside the bacterial host may elevate up to 1 mM under certain conditions^25^. Therefore, we have measured ATPase activities of wt HutD and the variants upon elevating ATP concentrations to 500 μM as well. All four mutants showed negligible ATPase activities even with an elevation of ATP concentration to 500 μM (Fig. 3a *right panel*). The increase of incubation time to 20 min also did not affect the ATP hydrolysis pattern of the mutants compared to wt HutD (Fig. 3b).

### Modelling of HutD and HutCD in OF and IF conformational states

Oligomeric states of NBD of ABC transporters and mechanism of ATP binding/hydrolysis differ significantly in the case of type-I and type-II importers. While in the case of the type-I importers dimerization of NBDs are influenced by interactions with permeases, type-II NBDs can form dimer independently^23^. Sequence comparisons proposed HutCD as a type-II importer (Fig. 1). Our biochemical results established that HutD forms stable functional dimer (that can bind and hydrolyse ATP) in the absence of the permease HutC (Fig. 2a–c). Therefore, we decided to investigate the dynamic nature of HutD dimers in the OF and IF states.

**Figure 4:**
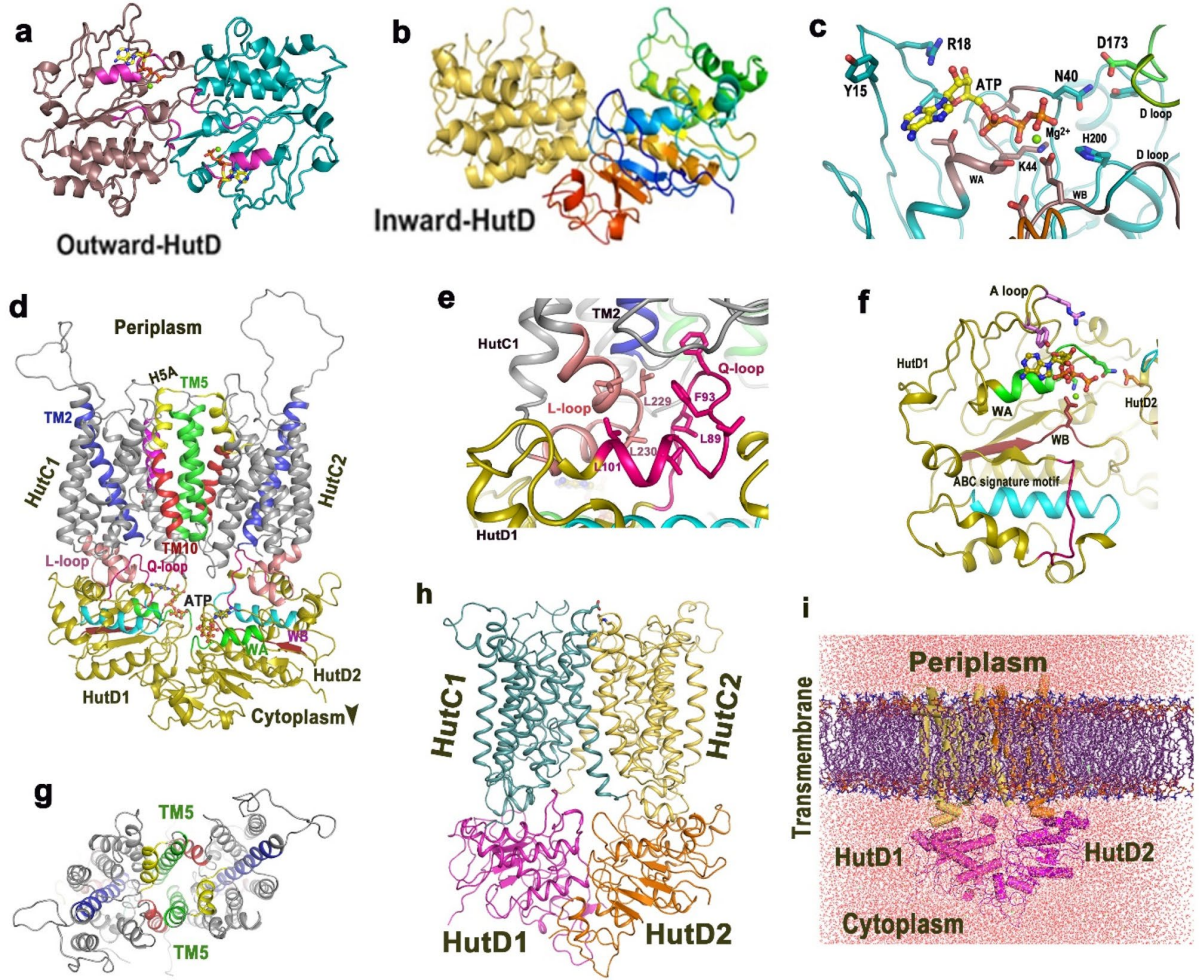
Models of HutD, HutC in different states and lipid embedded assembly. Ribbon representation of the models of (**a**) HutD dimer in the OF state, Nucleotide-binding domains (NBDs, dimer of HutD) are shown in brown and cyan, conserved Walker-A motif or P-Loop is shown in magenta of each NBD. Two ATP molecules are shown in stick; (**b**) HutD dimer in the IF state; (**c**) zoomed view of the docking of Mg^2+^-ATP in HutD OF dimer and related interactions; (**d**) Mg^2+^-ATP bound HutCD dimeric assembly in the OF state; zoomed view of different regions of HutCD are shown in (**e–g**). (**e**) Interactions between L-loop of HutC1 (salmon) with Q-loop of HutD1 (magenta); (**f**) disposition of binding motifs such as A-loop, Walker-A (green), Walker-B (maroon) around Mg^2+^-ATP. ABC signature motif is shown in cyan; (**g**) arrangements of transmembrane helices in HutCD-OF model is shown from the periplasmic side. TM5 helices of each TMD is shown in green, H5a in yellow; (**h**) Model of HutCD assembly in IF state. (**i**) Model of HutCD assembly embedded in solvated DMPC lipid bilayer.

The structures of the two type-II Heme importers, HmuUV of *Y. pestis* (PDB code: 4G1U) and BhuUV from *B. cenocepacia* (PDB code: 5B57), in two different conformational states, served as templates to prepare the models of HutD and HutCD in the OF and IF states. First, we prepared the models of HutD dimers in OF and IF conformations (Fig. 4a,b). Previous studies on type-II ABC transporters suggested that ATP hydrolysis mainly occurs in the OF state^26^. Therefore, knowledge-based docking of two Mg^2+^-ATP molecules was performed with HutD in OF state (Fig. 4a,c) where coordinates and binding mode of ATP in MJ0796 NBD dimer (PDB code 1L2T) was treated as template. Dimers of HutC in OF and IF states were also modelled in a similar manner. All models were prepared initially using ps2v2^27^ and later verified by Alphafold2^28^. HutCD assemblies in IF and OF states were then generated considering the assembly structures of HmuUV and BhuUV (Fig. 4d–i).

Altogether, we have prepared five sets of complexes: (1) Mg^2+^-ATP bound HutD dimer in OF state; (2) HutD dimer in IF state; (3) lipid bilayer embedded Mg^2+^-ATP bound HutCD assembly in OF state; (4) lipid bilayer embedded HutCD in IF state; (5) Heme bound lipid bilayer embedded HutCD in IF state. MD simulations, each of 1 μs, were carried out on all complexes with the Amber-18 and AmberTools-18 software packages^29^. We have used DMPC (1,2-dimyristoyl-sn-glycero-3-phosphocholine) molecules for constructing the bilayer and CHARMM-GUI^30^ was used to prepare the solvated protein-embedded lipid bilayer system before simulations. An embedded model is shown in Fig. 4i. However, the lipid bilayer and the solvents molecules were not shown in the subsequent figures to maintain clarity.

### Dynamics of HutD dimer in free and in HutC bound states

**Figure 5:**
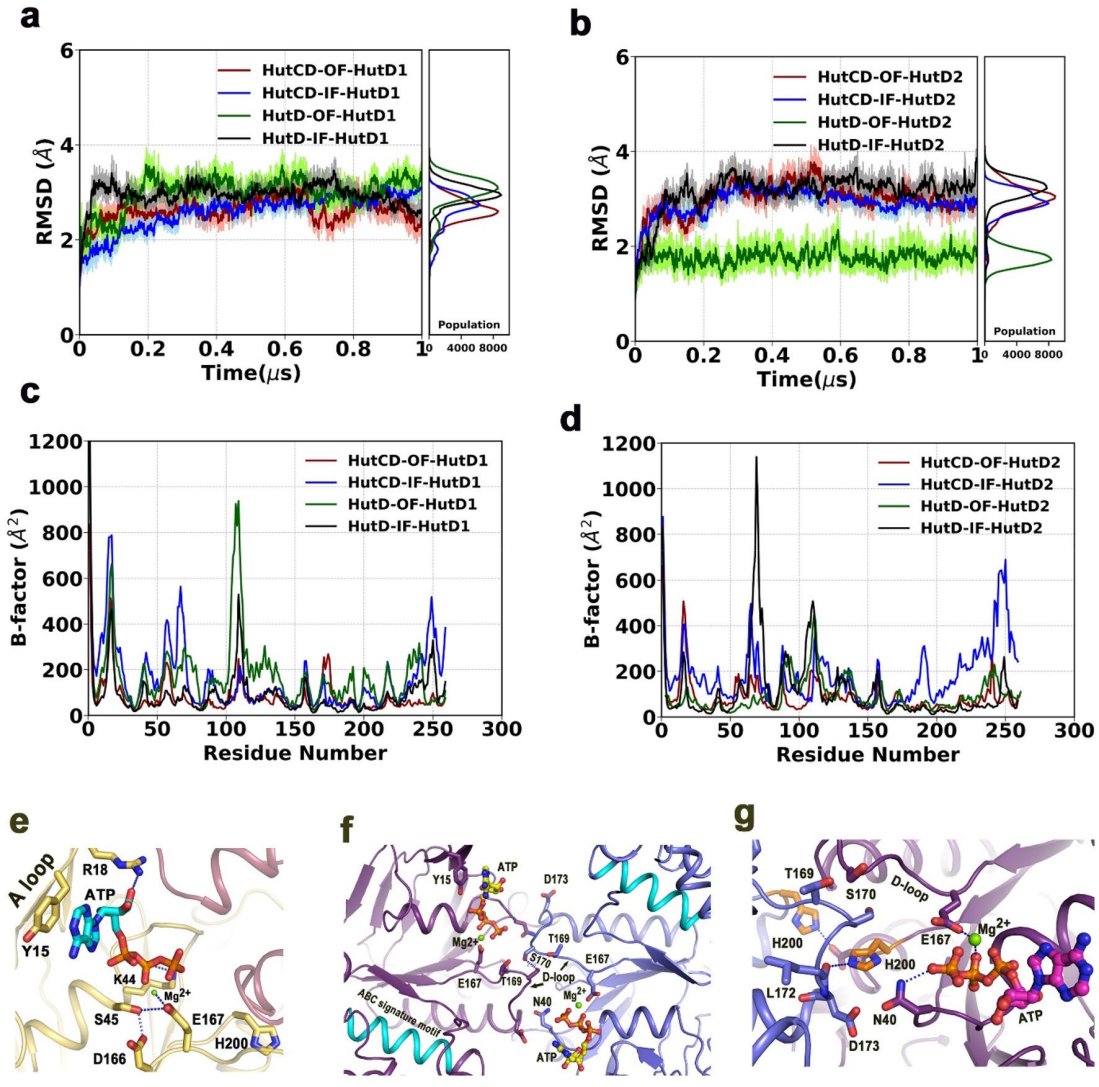
MD simulation results of HutD in the OF state. (**a,b**) Cα-RMSD values of HutD1 and HutD2 as part of HutD dimers and as part of HutCD assemblies in the IF and OF states; (**c,d**) B-factors of HutD1 and HutD2 as part of HutD dimers and as part of HutCD assemblies in the IF and OF states; (**e**) ATP binding in HutD OF at 200 ns showing involvement of the residues of A-loop, Walker-A and Walker-B motifs; (**f**) interaction between D-loops of two HutD monomers at 108 ns of simulation run on HutD dimer in the OF state; (**g**) disposition of H200 at 150 ns of the simulation run on HutD dimer in the OF state.

In order to understand the collective internal motions of HutD dimers in the OF and IF states, we have calculated root-mean-square deviations (RMSDs) of the Cα atoms from the snapshots of the trajectories up to 1 μs for the two chains of NBDs of HutD dimer and HutCD dimer (Fig. 5a,b). In all cases, RMSD values converged well to a constant value after approximately 75 ns. RMSD values of the two HutD protomers (named as HutD1 and HutD2) were plotted separately (Fig. 5a,b). The RMSD values for HutD1 of HutCD OF, HutCD IF, and HutD IF and OF were steady around 3.0 to 3.5 Å (Fig. 5a). However, HutD2 in OF state showed relatively lower RMSDs ranging around 2 Å (Fig. 5b). This indicates that NBDs acquire stability upon Mg^2+^-ATP binding, although asymmetric movements were observed between the monomers (Fig. 5a,b).

B-factors of the two chains of HutD were also plotted separately. The highest B-factors were observed for HutD and the B-factors were lowest for HutD of HutCD in ATP-Mg^2+^ bound state (Fig. 5c,d). HutD of HutCD IF state showed intermediate flexibility (Fig. 5c,d). Further analysis of B-factors of the two monomers of HutD showed asymmetry/dissimilar movements. Reduction in fluctuations in the residues 100–115 of HutD1 in HutCD indicates that not only ATP-Mg^2+^ but the TMDs also help in stabilization of the complex, because of the interactions between L-loop of HutC and Q-loop of HutD (Figs. 4e, 5c,d). The flexibility and asymmetric nature of the A-loop (residues 12–23, Fig. 1b) are also evident for all the states, which is more distinct in case of HutD1 compared to HutD2 (Fig. 5c,d). As evident from large B-factor values, high flexibility were observed in certain surface exposed regions of HutD (Fig. 5c,d).

### Interactions near ATP binding site of HutD dimer

Analysis of the simulation trajectory of HutD dimer in the presence of Mg^2+^-ATP bound OF state revealed that Y15 of the A-loop packs hydrophobically with the adenine base of ATP where R18 interacts with the ribose sugar of ATP (Fig. 5e). During dynamics, Mg^2+^ consistently remains bound to E167 of Walker-B and is poised to hydrolyze ATP (Fig. 5e,f). D166, the neighboring residue, interacts with S45 of Walker-A and thereby stabilizes the Walker-A conformation. K44 acts as the pivotal residue of Walker-A that interacts and stabilizes β- and γ-phosphates of ATP (Fig. 5e). These observations corroborate with abrogation of ATP hydrolysis of R18, K44, D166 and E167 to Ala (Fig. 3).

In the OF state, the D-loops of the two HutD monomers interact with each other at the dimeric interface which is mediated primarily by T169 and S170 (Fig. 5f). H200 also participates in dimerization (Fig. 5g). H200 of both the chains interacts with the D-loops belonging to the *trans* protomer (Fig. 5g). N40 is found to play a dual role. This residue is capable of interacting both with the γ-phosphate of bound ATP and D173 of the D-loop belonging to the *trans*-acting HutD (Fig. 5f,g). ATP binding was found to be assisted by ‘ABC signature motif’ in BtuCDF^26^. However, we have hardly observed any contribution of ‘ABC signature motif’ in ATP binding for HutCD (Fig. 5f).

### Dynamics of HutCD in the OF state

**Figure 6:**
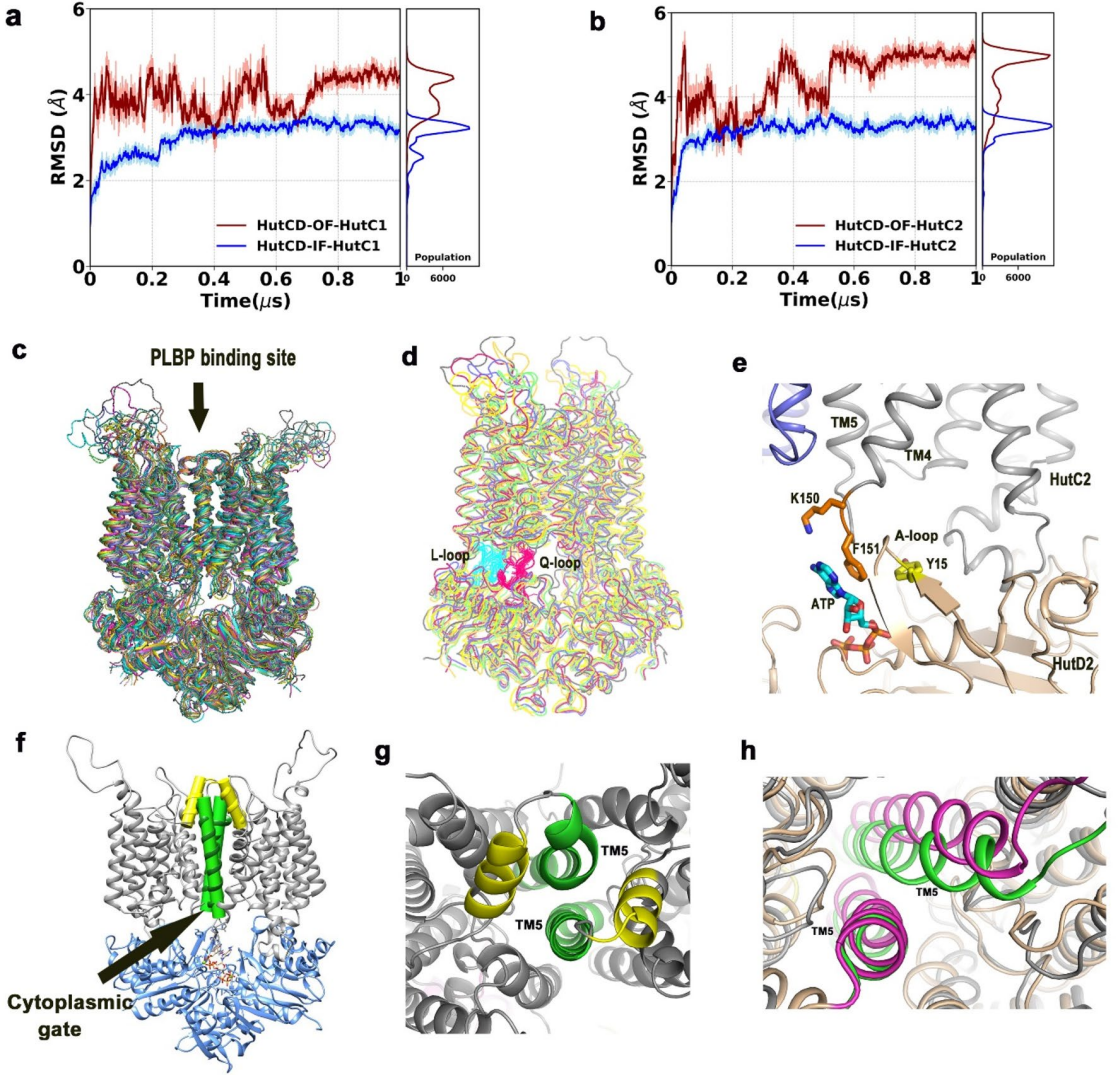
MD simulation showed OF to IF transition in HutCD. (**a,b**) Cα-RMSD values of HutC1 and HutC2 as part of HutCD assemblies in IF and OF states; (**c**) superposition of snapshots showing overall movement in HutCD OF state. PLBP binding site using an arrow; (**d**) superposition of snapshots showing proximity between L-loop (cyan) of HutC and Q-loop (pink) of HutD during dynamics; (**e**) snapshot of 150 ns shows interaction of F151 of HutC with ATP and Y15 of A-loop of HutD that causes cytoplasmic gate opening; (**f**) TM5 helices (green) of HutCD OF at the beginning of MD run; (**g**) zoomed view of TM5 helices (green) and H5a (yellow) of the beginning frame from periplasmic side; (**h**) superposition of snapshots of HutCD OF starting frame (green) and 150 ns (magenta) shows increased inter TM5–TM5 distance at 150 ns leading to cytoplasmic gate opening.

We observed that RMSDs of the two HutD chains of HutCD converged after 75 ns and remained steady around 2.5 Å and 3 Å respectively. To investigate the flexibility and conformational shift of TMDs in HutCD, we have calculated the RMSDs of HutC1 and HutC2 in OF and IF states from MD trajectories run up to 1 μs. RMSD plots of two TMD chains (Fig. 6a,b) indicated that the values converged after approximately 75 ns. While RMSD values of HutCs in the IF state remained stable around 3 Å (Fig. 6a,b), significant fluctuations were observed in the RMSDs of HutCs in the OF state, ranging between 4 and 5 Å. Overall superposition of the snapshots in OF state (ranging between 100 and 750 ns) demonstrated high flexibility of the loops exposed to periplasmic side and encompassing the PLBP binding site (aa 30–55 of HutC) (Fig. 6c). Although these loops of both HutC monomers were apparently in the upright conformation in the staring model (Fig. 4d), after dynamics they culminated in different conformations (Fig. 6c). In one HutC monomer, this loop region was found in upright conformation whereas in the other it acquired an inclined conformation (Fig. 6c). Asymmetric movement of this loop in two HutC monomers might be attributed to ease the accommodation of the PLBP.

### Synchronized conformational shift of HutD and HutC in ATP-Mg^2+^ bound OF state HutCD

Analysis of the simulation trajectory of HutCD OF state indicated that the interactions between the D-loops of the two HutD monomers are instrumental in the transmission of conformational shift to HutC. In HutCD, the distance between S170 of the SALD motif of the D-loops of the two monomers varied from 2.9 (103 ns) to 17 Å (606 ns) (Table 1). Upon proximity, the D-loops interacted with each other through H-bonding between S170 and/or T169 (Fig. 5f). N40 stabilized the γ-phosphate of ATP and interacted with D173 belonging to the ‘SALD motif’ of the D-loop of *trans*-acting protomer (Fig. 5f,g). However, this interaction was inversely related to inter HutD interactions through the D-loops, which is evident from Table 1. Upon proximity of the D-loops of the two monomers of HutD, N40 interacted with the γ-phosphate of ATP practically facilitating ATP hydrolysis.

**Table 1:** Random frames taken in order to see any proximities between S170 of HutD1:N40 of HutD2, N40 of HutD1:S170 of HutD2 and S170 of HutD1:S170 HutD2.

Trajectory of HutCD OF state (ns)	S170 of HutD1/N40 of HutD2 (Å)	N40 of HutD1/S170 of HutD2 (Å)	D-loop distance S170–S170
79	11.65	5.49	4.6
98	11.15	5.21	5.0
103	7.2	5.38	2.92
117	10.7	7.2	3.5
127	7.64	7.56	2.93
136	5.15	4.17	9.48
141	4.02	2.81	8.65
151	4.79	2.63	8.24
184	3.88	5.15	8.09
342	4.67	4.92	11.69
381	2.89	4.47	9.93
400	4.70	2.92	10.30
420	3.71	3.88	11.22
450	3.36	4.96	10.020
481	4.70	4.76	11.889
500	3.36	3.158	11.550
553	4.863	4.653	12.052
595	4.66	4.82	12.299
606	5.80	6.9	17.26

Available structures suggest that a cytoplasmic loop, called L-loop that is located between TM6 and TM7 helices of TMD, shares extensive contacts with the complementary groove on the NBD surface and is proposed to be critical in TMD-NBD communication^31^. Such interactions were evident in HutCD as well (Fig. 4d). Superposition of the snapshots, collected from 50 to 580 ns of the simulation run of HutCD, established retention of proximity and hydrophobic interactions between L-loop of HutC and Q-loop of HutD (Fig. 6d). The conserved Q85 of the Q-loop of HutD was also found to interact with the *cis*-acting D-loop. The conformational shifts in HutD dimer, caused by the position and the interactions of the D-loops, therefore, relayed to the L-loop of HutC through the Q loop of HutD. Trajectories of HutCD in OF state between 140 and 300 ns showed vigorous structural movements, and after 300 ns there has been a continuous loosening and widening of the overall structure, with concomitant dissociation of HutD1–HutD2 interface by distancing of D-loops (Table 1).

### Opening of the ‘cytoplasmic gate’

The ‘cytoplasmic gate’ that resides in cytoplasmic side of TM5 is known to be instrumental in heme translocation, as evident from the structures of HmuUV and BhuUV^13,14^. F151 that belongs to the loop between TM4 and TM5 of HutC located near cytoplasmic gate is found to be important in terms of transmission of the signal. At the beginning of simulation, this loop was 13 Å away from HutDs. During dynamics, F151 was seen to pack with adenine base of ATP and Y15 of the A-loop of HutD (Fig. 6e). Interestingly, the involvement of F151 in such hydrophobic packing is not consistently simultaneous in each HutC–HutD duo. In early stage of simulation, such as 80 ns to 150 ns, the interaction remained simultaneous in both HutC1–HutD1 and HutC2–HutD2. However, after 200 ns the interactions were not that consistent. Subsequently, F151 of HutC packs with Y15 of HutD but adenine base of ATP remains sufficiently away from those residues. In these stages of the trajectory, such interaction was observed in one HutC–HutD while in the other the TM4-TM5 loop was away from the ATP binding site. At the beginning of MD simulation ‘cytoplasmic gate’ was closed (Fig. 6f) and as expected with OF structure, periplasmic side of the heme uptake channel was open (Fig. 6g). During the progress of the simulation run, packing of F151 of HutC with ATP and Y15 of HutD increases the distance of TM5 in cytoplasmic side essentially opening the cytoplasmic gate (Fig. 6h). A superposition of the snapshots at 150 ns on that of frame 1 depicts such opening of the ‘cytoplasmic gate’ (Fig. 6h). This conformational shift made us hypothesise that ATP binding and hydrolysis facilitates opening of the ‘cytoplasmic gate’ for heme uptake.

### Occluded state is observed in the simulation of ATP-free IF state of HutCD

**Figure 7:**
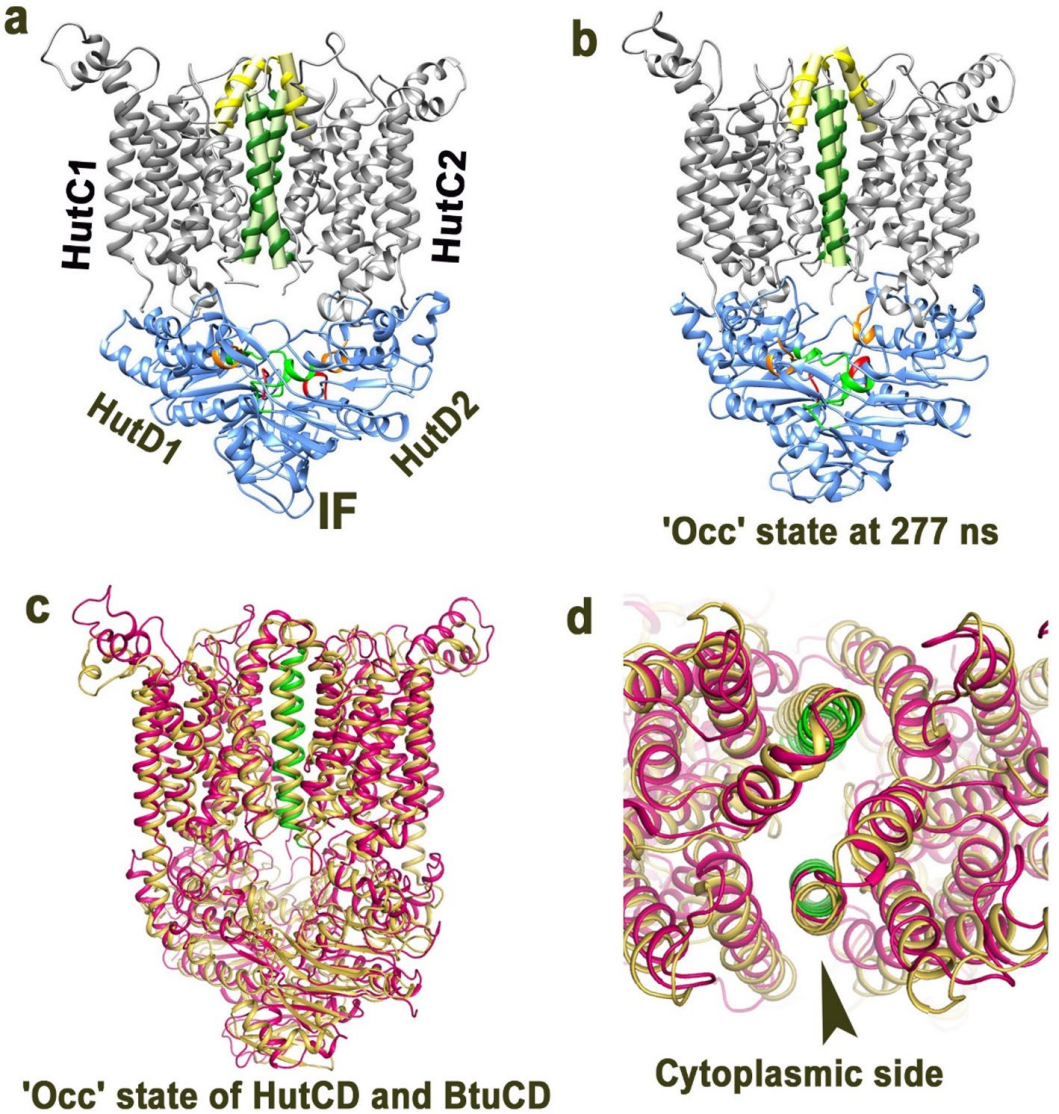
IF state of HutCD culminated to occluded state. (**a**) Ribbon representation of HutCD-IF model at 1 ns, showing axes of H5A (yellow), and TM5 (green), of each TMD in HutC dimer. (**b**) HutCD IF turned to ‘occluded’ state, snapshot taken at 277 ns from the unbiased MD study. Both in (**a,b**), D-loop, Walker-A and ‘ABC signature motif’ of HutD are shown in red, green and orange respectively. (**c**) Superposition of the ‘occluded’ state snapshot of HutCD IF at 277 ns on the ‘occluded’ state crystal structure of BtuCD (PDB code: 4FI3) (**d**) Zoomed view of (**c**) from cytoplasmic side showing similar orientation of TM5 helices (shown in green in case of HutCD).

As expected, the HutCD IF model showed inward facing orientation of the TM5 helices (Fig. 7a). Overall RMSD values of HutC and HutD dimers remained around ~ 3 Å during simulation. Throughout the dynamics, the periplasmic side remained tightly sealed by salt bridge interactions between D182 and R186 along with hydrophobic packing between L185 of two H5a helices. Although the crossing angle of H5a–H5a was little lower (50.8°) than the structure of BhuUV (73.1°), like BhuUV, the position of H5a helices remained almost unchanged during dynamics. Notable transition in the orientation of the TM5 helices was observed from 277 ns. At this stage and onward, drastic reduction in the inter TM5–TM5 crossing angle occurred from 23.9° to 6.4° (Fig. 7b). Superposition of the snapshots of HutCD IF, collected at ~ 277 ns, with the occluded ATP bound crystal structure of vitamin B12 importer BtuCD (PDB Code: 4FI3) showed a RMSD of 3 Å (Fig. 7c). Maximum differences were observed among the NBDs, while orientation of the TM5 helices of the two structures were outstandingly similar to each other (Fig. 7c,d). The crossing angle/distance of TM5 axes was 6.4°/16.2 Å in case of HutCD, and that of the ATP bound occluded structure of BtuCD is 11.2°/19.0 Å (PDB Code: 4FI3). These observations suggest that unbiased MD simulation of the HutCD in IF state leads to gradual transition to the ostensible occluded state from ~ 277 ns by exclusive movement of the inter TM5 helices (Fig. 7).

The NBD domains of HutCD IF state remained oriented throughout dynamics, where the main flexible portion was the A-loops (Fig. 5c). In IF state, the *trans*-acting ‘ABC signature motif’ was found near the Walker-A, although the distance was not equal in case of the two protomers. Gradual proximilty of Walker-A and *trans*-acting ‘ABC signature motif’ with the progress of simulation provided compactness to the NBD dimer. The D-loops at the NBD interface were less flexible than the D-loops of Mg^2+^-ATP bound OF state HutCD. In contrust to the OF state where D-loops of HutDs were found to interact, in the IF state, inter D-loop distance was ranging from 6 to 11 Å in most of the frames.

### Identification of heme release pathway in HutCD IF state

**Figure 8:**
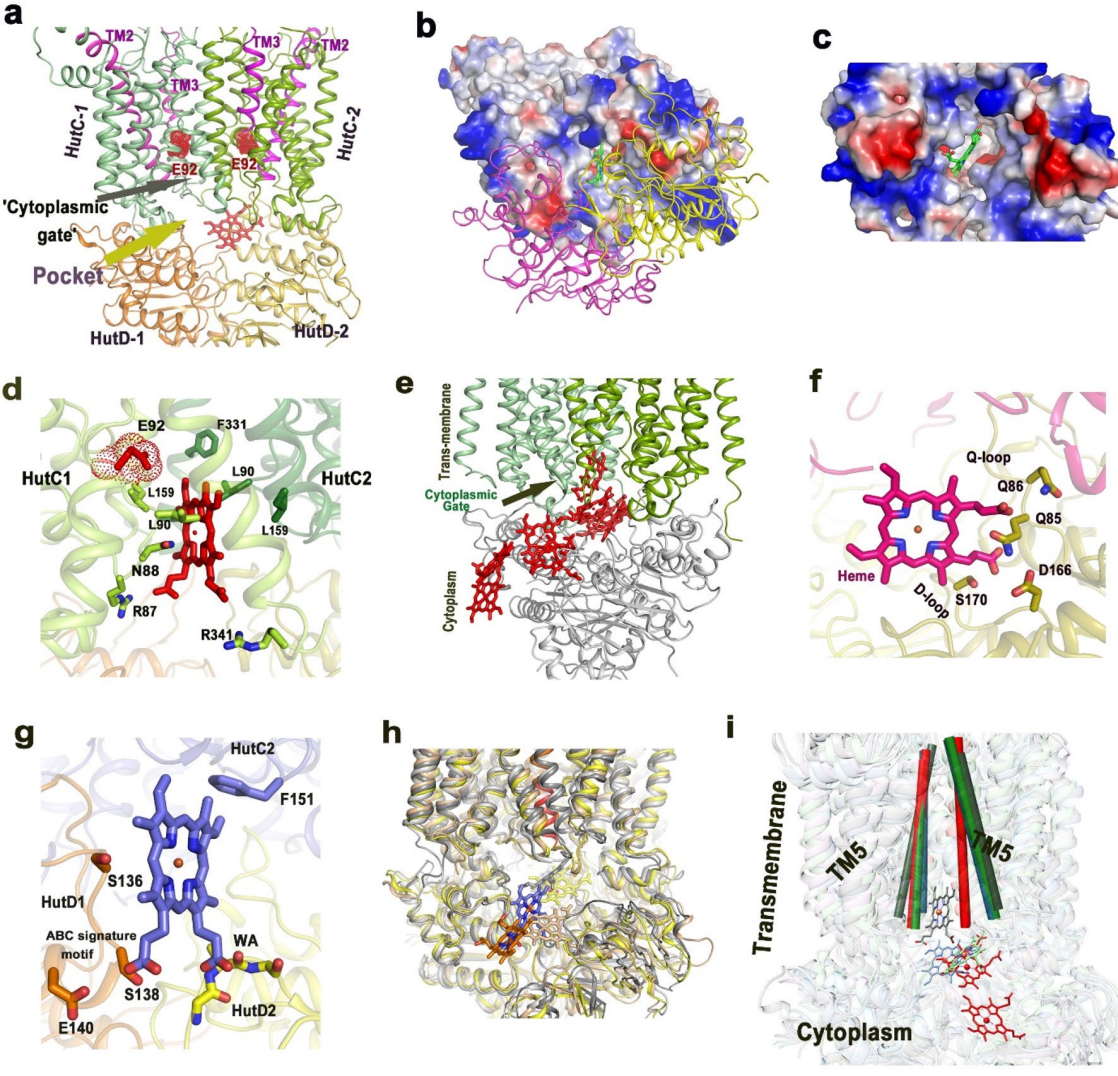
Heme release pathway from ‘cytoplasmic gate’ to cytosol. (**a**) Ligand release pocket between the two dimers, HutC1–HutD1 and HutC2–HutD2 and cytoplasmic gate are shown here.TM2, TM3 of each HutC monomer are shown in pink while E92, belonging to TM2–TM3 loop is shown in red; (**b**) Docking of Heme near cytoplasmic gate of HutCD IF; HutC dimer is shown in electrostatic surface representation whereas HutD dimer is shown in cartoon representation. Docked heme is shown as green sticks; (**c**) Zoomed view showing docked heme at ‘cytoplasmic gate’ of HutC dimer of HutCD in the IF state. HutD dimer is not shown here for clarity; (**d**) Zoomed view showing docking of heme near ‘cytoplasmic gate’ and its interactions with HutC residues; (**e**) Release pathway of heme from HutCD; (**f**) Snapshot at 133 ns shows interaction of Heme with Q-loop and D-loop residues of HutD; (**g**) Interaction of heme with F151 of HutC and S136, S138 of ‘ABC signature motif’ of HutD on the verge of release to cytosol; (**h**) Superposition of snapshots of HutCD IF at 85, 121, 410, 468 ns. HutC and HutD subunits undergo limited fluctuations during release of heme. (**i**) Zoomed view of superimposed snapshots reveals noticeable conformational shift ofTM5 helices (shown as cylinder) at 410 and 468 ns (red) especially in one TMD of HutC dimer compared to 1 (grey), 85 (green), 121 (blue) ns, leading to ‘occluded’ state during heme release.

Naoe et al. stated that D112 of the TM2–TM3 loop of the permease BhuU of *B. cenocepacia* is the only charged residue exposed to the inward facing heme translocation pathway of BhuUV and plays critical role in the heme translocation process, because mutation of this residue to the hydrophobic residues impaired transport activity^14^. Corresponding residue of HutC is E92 and as expected, this residue of both the HutC monomers are exposed to the heme translocation channel in the IF model of HutCD to impart polarity (Figs. 1a, 8a). We, therefore, intended to investigate the binding pattern and movement of heme, after it reaches the ‘cytoplasmic gate’. For that reason, heme was docked near the cytoplasmic gate of inward facing HutCD using Autodock Vina^32^ (Fig. 8b,c). The hydrophobic site was made of L159 and V163 of TM5 along with L90 of TM2–TM3 loop. One propionate group was hydrogen bonded with R87 of the TM2–TM3 loop (Fig. 8d).

Soon after beginning of simulation, the heme molecule moved to the pocket between two HutC-HutD dimers (Fig. 8e–g). At this stage, the propionate group was interacting with the backbone NH groups of the Q-loop residues L89 and T90 of HutD1. Heme stayed inside this pocket ‘flip flopping’ around for a significant period of time. Subsequently, heme was found to interact with N40 of Walker-A and the D-loop residues of HutD. Polar interactions of the propionates of heme with Q86, S88, T90 of HutD (HutD1) were steady from 350 to 430 ns (Fig. 8f). Additionally, F151 of HutC was found to pack with heme (Fig. 8g). Notably, F151 of HutC was crucial for cytoplasmic gate opening during ATP hydrolysis by HutCD in OF state. The pocket between two HutC-HutD dimers started squeezing around 455 ns. Around 465 ns the heme changed orientation and moved near the other HutD monomer (HutD2) where the propionates were interacting with S138 of HutD2 (Fig. 8g). Heme was on the verge of release to cytosol around 472 ns where heme was in contact with S138 of the ‘ABC signature motif’ of HutD2. At 473 ns, the propionate group of heme had polar interaction with S136 followed by H128 and the molecule is finally released to the cytosol (Fig. 8e–i). A detailed list of interactions between HutCD and heme is given as Table S2.

During the process of heme release, no significant structural change occurred in the TMDs except orientation of TM5 (Fig. 8h). After 400 ns, the TM5 helices started acquiring an occluded conformation which was prominent around 460 ns (Fig. 8i). Attainment of occluded state is evident from the superposition of the snapshots collected at 85, 121, 410 and 468 ns (Fig. 8i). Here, conformation of TM5 remained unchanged in HutC1 where cytoplasmic side of TM5 of HutC2 shifted about 5 Å towards central axis of the translocation channel (Fig. 8i).

## Discussion

Dimerization of the NBDs in ABC importers of prokaryotes, whether nucleotide driven or independent, is indispensable, since this is a vital early event of ligand translocation. Our results authenticated HutD as a type-II ABC importer, since it forms dimer even in the absence of the permease HutC (Fig. 2a–c). Based on the sequence comparisons, Y15 of the A-loop of HutD was predicted to pack with the adenine base of ATP (Fig. 1b). Substantial fluorescence quenching in the presence of AMP.PNP established significant binding of ATP with HutD in 1:1 stoichiometry with the involvement of Y15 (Fig. 2d–f). While wt HutD was an efficient ATPase, R18A, K44A, D166A and E167A turned out as loss-of-function mutants (Fig. 3a,b). Abrogation of ATPase activities in the mutants together with modelling, docking and MD simulations results suggested that R18 of the A-loop and K44 of Walker-A of HutD stabilize the ribose sugar and the β-phosphate of ATP respectively and hence, ATP binding is compromised upon removal of those side chains (Figs. 1b, 3a,b, 4c, 5e). Based on the sequence analysis (Fig. 1b) we presumed that D166 and E167 are crucial Walker-B residues for conducting ATP hydrolysis event. Our observations demonstrated that E167 is implicated in Mg^+2^ binding for ATP hydrolysis while neighbouring D166 interacts with S45 of Walker-A to facilitate ATP binding by stabilizing Walker-A. Rather, a network of polar interactions was observed between S45 of Walker-A and D166, E167 of Walker-B as evident from the snapshot of MD simulation (Fig. 5e). Mutation of D166 to Ala presumably generates more space for E167 side chain and at the same time turns E167 more flexible because of impaired polar interactions stated above, eventually disturbing Mg^+2^ binding/ATP hydrolysis.

MD simulation trajectory of HutD in ATP bound state depicted that D-loops of the two HutD monomers interact at the dimeric interface and H200 critically contributes to the dimerization by interacting with the *trans*-acting D-loop (Fig. 5g). MD simulation results further suggested that ATP binding to HutD does not need any assistance from the *trans*-acting ‘ABC signature motif’ (Fig. 5f), like it was observed before in the crystal structure of BtuCD-F^26^. Rather, in HutCD, ‘ABC signature motif’ interacts with the heme during its ejection to the cytosol. Presumably, the translocation of bigger ligand vitamin B12 requires a wider channel compared to heme (HmuUV structure) and the NBDs need to dimerize accordingly. The difference in ATP binding mechanisms might be attributed to the differential inter dimeric interactions of BtuD and HutD which are essential for the ligand- and species-specific transportation pathways.

Conformational shifts of certain transmembrane helices, especially the movable helices TM5 and H5a regulate the ligand import processes through ABC importers. Substrate translocations through the translocation channels of type-I ABC transporters MalK of *E. coli* are dictated by ‘tweezer’ like or ‘twisting’ motions of the NBDs^33,34^. For type II importers, substrates translocation by open/closure of the cytoplasmic gate is thought to occur via ‘peristaltic’ motions of the TMDs^16^. In HutCD, a rotational motion occurs in the TMDs in synchronous manner with the inter NBD D-loop interactions (Table 1). The more the separation of the inter HutD D-loops, the more rotation of the NBDs which is then transmitted to the TMDs. The distance between the two HutC–HutD dimers was found to be altered accordingly. However, asymmetry has been observed in the interface interactions between HutC and HutD for each HutC–HutD duo.

The interaction of the SKF^151^ loop of HutC, located between TM4 and TM5 near the cytoplasmic gate, with ATP and the A-loop (Y^15^GSR) of HutD is one of the most prominent facts that tie the event of ATP hydrolysis with the conformational change in HutCs to facilitate ligand translocation (Fig. 6e). Packing of F151 with the adenine base of ATP and Y15 of the A-loop of HutD leads to the widening of the cytoplasmic gate where the periplasmic side of the TM5 helices remained practically unchanged (Fig. 6e,h). Interestingly, in the later stage of the dynamics (~ 400 ns and beyond), which might be treated as post hydrolysis phase, continuous relaxation of the assembly was observed and, in this situation, TM5 helices prefers to acquire relatively parallel conformation. Inward facing assembly was also culminated to an occluded conformation, similar to that observed before for vitamin B12 importer BtuCD (Fig. 7c,d). We, therefore, hypothesize that the occluded state is the most preferred conformation of HutCD.

H5a was seen to play a key role as a gating helix to forming the interaction site for the PLBP, BhuT in the case of BhuUV-T assembly^14^. Also, mutation of R176 in H5a of HmuU was reported to decrease affinity to HmuT and decrease heme-transport activity^13^. Although TM5 helices showed visible transition near the cytoplasmic gate, conformation of H5a helices, located at the periplasmic side, practically remianed unchanged during the dynamics. We, therefore, hypothesize that the movement or change in orientation of H5A helices are most likely PLBP dependent, and subsequent separation of the PLBP from the TMDs may result in the transition of orientations of the H5A helices.

**Figure 9:**
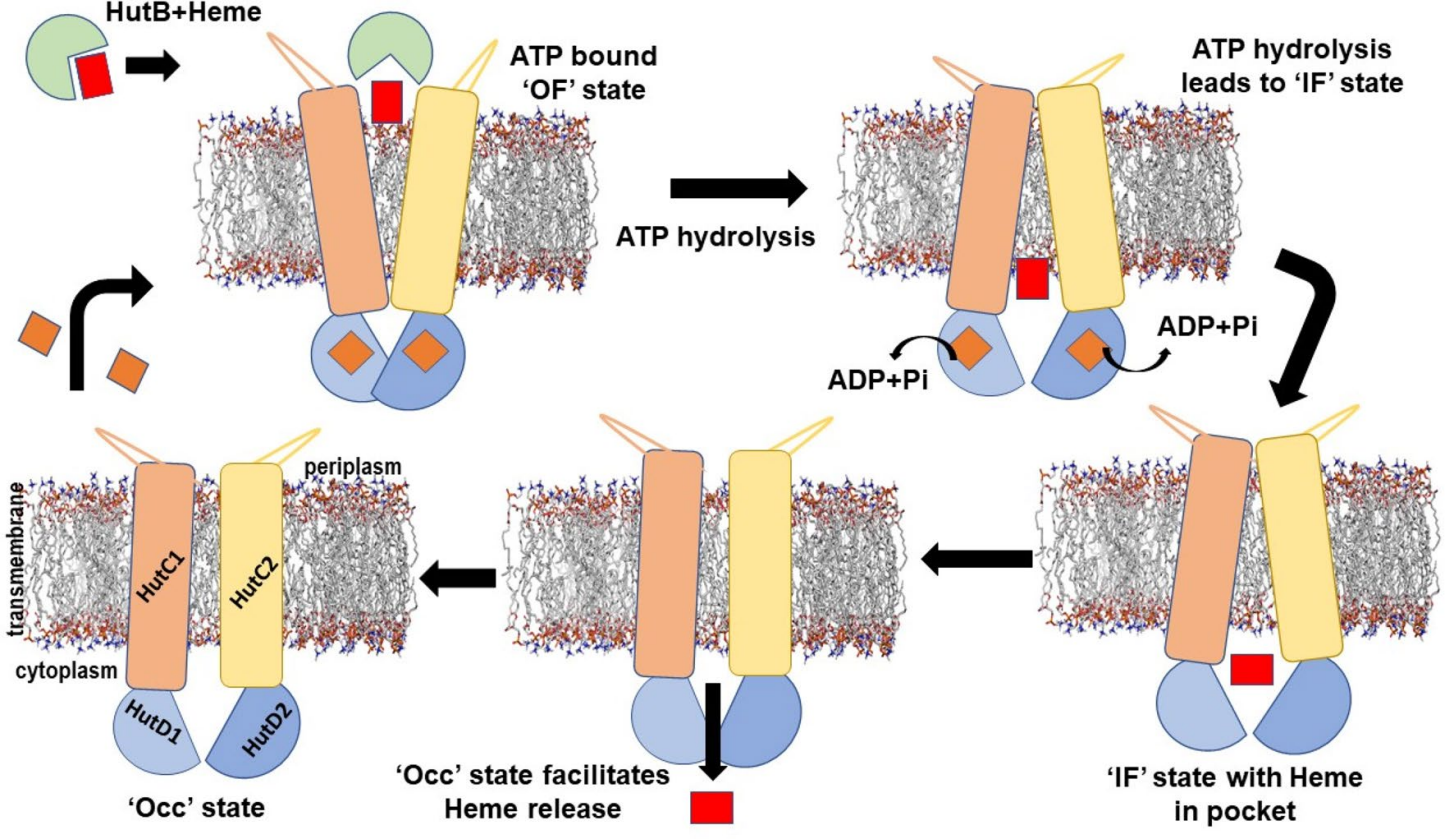
Proposed reaction mechanism. Involvement of TM4–TM5 loop in ATP hydrolysis causes cytoplasmic gate opening (IF state) easing heme release to the inter-dimeric pocket. Heme release to cytoplasm takes place in the absence of ATP. Formation of occluded state constricts the pocket and releases heme.

The heme release mechanism has been deciphered for the first time which was unknown so far. Our extensive simulation showed that the release of heme is primarily dictated by the interaction of the propionate groups with the polar residues of HutC–HutD dimeric pocket (Fig. 8f,g). Interaction of heme with the residues of D-loop, Walker-A, Q-loop and ‘ABC signature motif’ (Fig. 8f,g, Table S2) further suggested that heme release event takes place in the absence of ATP. Because the presence of ATP would have hindered involvement of these motifs in the heme release process. As observed in case of heme-free HutCD in the IF state, heme bound form also acquired occluded state at the verge of heme release. Parallel conformation of TM5 helices in the occluded state probably leads to the squeezing of inter-dimeric pocket which eventually facilitates the heme release (Fig. 9). Hence ABC importers like HutCD prefers to acquire occluded conformation before starting the next cycle of ATP hydrolysis dependent heme translocation (Fig. 9).

Type-II importers HmuUV, BhuUV or BtuCD were predominantly in different states while trapped in the crystal lattice^13,14,26^. Notably, Y15 of HutD or F151 of HutC, which stack with the adenine base of ATP leading to conformational changes in HutCD assembly, are not conserved in HmuV or BhuV (Fig. 1b). Although similar hydrophobic residues are observed in HmuUV in the close vicinity of the respective residues, BhuUV is quite different in this regard (Fig. 1). Furthermore, S170, the crucial D-loop residue of HutD is replaced by Ala in BhuV (Fig. 1b) generating a possibility of compromised D-loop interactions. Collectively, the observations intend us to believe that although there is a common minimum procedure to internalize a specific nutrient, detailed mechanism of uptake is primarily dictated by the requirement of that specific nutrient in the respective species.

## Methods

### Cloning, site-directed-mutagenesis, overexpression, and purification

Chromosomal DNA of *V. cholerae* strain O395 was used as the template to amplify the region encoding Full length HutD of 259 aa (Accession code: A0A0H3ADP8). The 780 bp HutD PCR amplicon and the pET28a^+^ vector (Novagen) with *NdeI* and *BamHI* restriction sites were ligated using T4 DNA ligase and the appropriate clones were selected using *E. coli* XL1-Blue cells with kanamycin resistance. The cDNA of HutD (amino acids 1–259) was PCR amplified and was cloned in pET28a^+^ within *NdeI* and *BamHI* restriction sites. The recombinant protein with N-terminal His_6_-tag was overexpressed in BL21 (DE3) by IPTG induction and purified by Ni–NTA affinity chromatography. The construct was verified by restriction digestion analysis and commercial DNA sequencing. The mutants R18A, K44A, D166A and E167A were prepared by two-step PCR amplification using previously cloned WT HutD plasmid as template. These mutant amplicons were also cloned in pET28a^+^ vector using *NdeI* and *BamHI* as restriction sites. Sequences of the variants were verified by commercial sequencing. These recombinant proteins with N-terminal His_6_ tag were overexpressed in *E. coli* BL21 (DE3) cells in the presence of IPTG.

Overexpression and purification of recombinant proteins were performed as per protocol described by Dey et al.^24^. In brief, 10 ml of LB broth, supplemented with kanamycin, was inoculated by a single colony, and grown by overnight shaking at 310 K. 1 l LB broth with kanamycin was inoculated with 10 ml of the overnight culture and the culture was grown further at 310 K until the OD_600_ reached 0.6. The cells were induced by 1 mM IPTG and grown at 310 K for another 3 h. The cells were then harvested at 4500×*g* for 20 min at 277 K and the pellet was resuspended in 10 ml ice-cold lysis buffer-L (having 50 mM Tris–HCl pH 8.0, 300 mM NaCl, 5 mM MgCl_2_, 10% (v/v) glycerol). PMSF (1 mM) and lysozyme (1 mg/ml) were added to the resuspended solution and it was lysed by sonication on ice. The cell lysate was then centrifuged at (12,000 RPM for 45 min) at 277 K. The collected supernatant was applied onto a nickel–nitrilotriacetic acid (Ni^2+^-NTA) affinity chromatography media (Qiagen) that was previously equilibrated with buffer-L. The 6× His-tagged recombinant proteins were eluted with 50–200 mM imidazole gradient. Subsequently, imidazole was removed from proteins through buffer exchange using Amicon centrifugation units. The wt HutD was concentrated up to 300 μM. All mutants were purified using the buffer containing 50 mM Tris–HCl pH 8.0, 300 mM NaCl, 10% (v/v) glycerol with the same protocol and concentrated up to 250 μM. The homogeneity of the purified proteins was checked using SDS-PAGE with 12% polyacrylamide concentration.

### Cross-linking experiments

Crosslinking experiments had been carried out with WT HutD and WT HutD in the presence of non-hydrolysable ATP analogue AMP-PNP using Glutaraldehyde (Sigma-aldrich) as crosslinker. HutD was purified in cross-linking buffer (50 mM Na-Phosphate pH 8.0 and 150 mM NaCl) followed by incubation with AMP.PNP (20 mM) on ice for 1.5 h. Increasing amounts of glutaraldehyde were added to the mix to a final concentration range of 0.0025–0.04%. The reaction mixture was further incubated at room temperature for 15 min. Crosslinking reactions were quenched by adding 50 mM Tris–HCL pH 8.0, followed by incubation at 97 °C for 10 min in standard SDS loading dye. Reaction products were separated by electrophoresis and analysed on 10% SDS-PAGE polyacrylamide gel.

### Gel filtration assay

Size Exclusion chromatography experiments were performed in a Superdex 200 Increase 10/300 GL using AKTA purifier (cytiva). Blue dextran was used to identify the void volume. The column was further calibrated with standard molecular weight calibration kit (GE Healthcare) containing Ferritin (440 kDa), Aldolase (160 kDa), Ovalbumin (45 kDa) and Lysozyme (14.3 kDa). The standard graph was prepared against relative elution volume (V_e_/V_o_) in X-axis [where V_e_ is the elution volume and V_o_ is the void volume] and the log molecular weight in Y-axis. For the AMP.PNP bound state, 250 µM HutD was preincubated for 1.5 h at 4 °C with 500 µM AMP.PNP in the same buffer-L. For analysis of HutD and HutD with AMP.PNP, in each run, 500 μl of protein was injected to the SEC column pre-equilibrated with buffer containing 50 mM Tris–HCl (pH 8.0), 300 mM NaCl, and 5 mM MgCl_2_ and ran at a flow rate of 0.5 ml/min. The peak fractions were collected and analysed in 12% SDS-PAGE.

### Fluorescence quenching study

As per protocol described by Agarwal et al.^22^, fluorescence measurements were carried out in Hitachi F-7000 spectrofluorometer, using quartz cuvettes of 1 cm path length. Changes in fluorescence of tryptophan and tyrosine residues were measured at an excitation wavelength of 280 nm and the emission spectra were recorded between 295 and 400 nm. Sole contribution of Trp residues upon AMP.PNP binding was also monitored with an excitation at 295 nm and emission between 308 and 400 nm. In both the cases, slit widths was kept consistently at 5 nm. All reactions were carried out at 298 K. The reactions were performed in a buffer containing 50 mM Tris–HCl (pH 8.0) and 300 mM NaCl. Equilibrium titration of HutD was carried out with AMP-PNP as ligand and the changes in fluorescence emission intensity were measured in the presence of increasing concentration of ligand. The concentration of HutD was 2.5 μM and ligand concentrations varied from 0 to 2 mM.

The binding stoichiometry was determined using the protocol described by Mani et al.^35^. The plot of log(F_0_ − F)/(F − F_∞_) against log [AMP-PNP], where F_0_, F, and F_∞_ are the fluorescence intensities of HutD alone, HutD, in the presence of various concentrations of AMP-PNP, and HutD saturated with AMP-PNP, respectively, yielded a straight line whose slope was a measure of the binding stoichiometry. The dissociation constant, K_d_ was determined using nonlinear curve fitting analysis as per Eqs. (1) and (2). All experimental points for the binding isotherms were fitted by the least-squares method:

1 $$K_{{\text{d}}} = \, \left\{ {\left[ {C_{0} {-} \, \left( {\Delta F/\Delta F_{\max } } \right) \cdot C_{0} } \right] \cdot \left[ {C_{{\text{P}}} {-} \, \left( {\Delta F/ \, \Delta F_{\max } } \right) \cdot C_{0} } \right]} \right\}/\left\{ {\left( {\Delta F/ \, \Delta F_{\max } } \right) \cdot C_{0} } \right\},$$

2 $$C_{0} \cdot \left( {\Delta F/\Delta F_{\max } } \right)^{2} {-} \, \left[ {\left( {C_{0} + C_{{\text{P}}} + K_{{\text{d}}} } \right) \cdot \left( {\Delta F/\Delta F_{\max } } \right)} \right] \, + C_{{\text{P}}} = \, 0.$$

where C_0_ and C_p_ denotes the input concentrations of the ligand and VcHutD respectively. Δ*F* is the change in fluorescence intensity at 334 nm (λ_ex_ = 280 nm) for each point of titration curve and Δ*F*_*max*_ is the same parameter when ligand is totally bound to the protein. A double-reciprocal plot of 1/Δ*F* against 1/(C_p_ − C_0_), as shown in Eq. (3) was used to determine the Δ*F*_*max*_*.*

3 $${1}/\Delta F = { 1}/\Delta F_{\max } + {\text{ K}}_{{\text{d}}} /\left[ {\Delta F_{\max } \left( {C_{{\text{P}}} {-}C_{0} } \right)} \right].$$

Δ*F*_max_ was calculated from the slope of the best fit line corresponding to the above plot^22^. All experimental points for the binding isotherms are fitted by least square methods using OriginPro 8.0 software.

### ATPase assay

ATPase activities of purified HutD and its mutants R18A, K44A, D166A and E167A were determined spectrophotometrically using malachite green assay by measuring the release of inorganic phosphate (Pi). As per protocol described before in Dey et al.^24^, each reaction mixture with final protein of 2.5 µM and buffer containing 50 mM Tris–HCl (pH 8.0), 300 mM NaCl, and 5 mM MgCl_2_ were incubated with ATP (Sigma Aldrich), of concentration ranging from 100 to 500 μM at 298 K. Malachite Green solution was freshly prepared by adding 0.44 g of Malachite green in 0.3 M H_2_SO_4_, 2.5 ml of 7.5% ammonium molybdate, and 0.2 ml of 11% Tween 20 to a final volume of 10 ml. 200 μl of aforesaid solution was added to the reaction mixture to a final volume of 1 ml. The absorbance was measured at 630 nm within 5 min of adding the colouring reagent. Pi standard curve was prepared by using KH_2_PO_4_ and plotting OD_630_ against release of Pi using OriginPro 8.0 software^24^. The total Pi released by each protein upon ATP hydrolysis was obtained from the standard curve. Each protein was checked with Malachite green dye without ATP to measure the contaminant inorganic phosphate if any, and the minor absorbance thus obtained at 630 nm was subtracted from the absorbance produced by that protein upon hydrolysis of added ATP. The ATP hydrolysis without protein has served as another negative control to nullify the effect of the contaminating inorganic phosphate with ATP. All the experiments were minimally performed in triplicate.

### Docking

Docking of heme with HutCD in the IF state has been performed using AutoDock Vina^32^ software. Coordinates of heme was retrieved from Docking of Heme molecule in the pocket of HutCD was performed using a grid box of dimension 65 Å × 40 Å × 52 Å centered at (− 29, + 14, + 15) Å.

### Building the lipid-protein system

The principal axis of the HutCD complex was aligned with the Z-axis, while the lipid bilayer spanned the XY plane. The HutCD complex was translated by 20 Å along the Z-axis to correctly place the TMD part within the hydrophobic region of the bilayer such that the polar head groups and hydrophilic lipid trails make contact with the specific residues of the transmembrane protein^36^ The multicomponent complex was enclosed in a tetrahedral box (approximate dimensions: a = 120 Å, b = 120 Å, c = 170 Å) and solvated in explicit water with a minimum water thickness of 22.5 Å on the top and bottom of the complex. A suitable number of neutralizing ions (Na^+^ or Cl^−^, depending upon the cases) were added by replacing solvent molecules using the Monte Carlo method. The number of DMPC molecules in a leaflet was in the range of 180 to 210, depending upon the protein being embedded (Fig. S3). The number of DMPC molecules on the upper and lower leaflets was slightly different (the maximum difference was 10). The area per lipid was calculated^37^ by multiplying the sides of the simulation box in the XY-plane and dividing it by the average number of lipid molecules in a leaflet (Fig. S4). The coordinate of the solvated multicomponent system was saved in PDB format and was modified afterward to make it consistent with the AMBER naming scheme.

### Force field for the ligands

All atom force field (FF) for the ATP cofactor was taken from the AMBER parameter database^38^ maintained by the Bryce group. ATP molecules were first made fully deprotonated and then hydrogens were added using “reduce” available in the Amber Tools. All the phosphate atoms in ATP were deprotonated and the total charge was − 4*e*. The atom names in ATP were also changed to match the naming convention of this FF. We used antechamber^39^ and MCPB.py^40^ to create the FF for the heme group. In the heme group, the iron metal coordinates to four nitrogen atoms. We have used a bonded model^41^ where Fe is bonded to four sp3 hybridised nitrogen. The antechamber program was used with the AM1-BCC charge method^42^ to assign charge by treating heme as having a charge of − 4*e* and the atoms were treated with GAFF atom types^43^. The procedure we followed to treat the heme group within the AMBER software package is described with great detail in one of the Amber tutorials^41^.

### MD simulation steps and parameters

Unbiased MD simulation in this work was carried out using pmemd.cuda, the GPU version (10) of MD engine within the Amber package. The protein backbone and side chains were treated with the FF parameters from ff14SB^44^, and lipid14 FF^45^ was used for the DMPC bilayer. All the simulations were carried out in explicit solvent with the three-point transferable interatomic potential (TIP3P) for waters^46^. In simulations involving only protein (i.e., the cases where bilayer was absent), a truncated octahedron box was used for solvation such that the edge of the box was at-least 10 Å away from the protein. A suitable number of Na^+^ or Cl^−^ ions were added to neutralize the solvated system. Periodic boundary condition was applied on all the sides of the solvated box. Bonds containing H-atoms were restrained using the SHAKE algorithm^47^ which enabled us to use a time step of 2 fs for the integrator. The pressure and temperature were controlled using a weak coupling to an external bath. The solvated system was coupled to a Berendsen barostat^48^ with a relaxation time of 2 ps, for maintaining a constant pressure of 1 atm. The particle mesh Ewald summation^49^ was employed for calculating the electrostatic interactions part of the total Hamiltonian. We applied a distance cutoff of 10 Å for calculations of the long-range electrostatic interactions. The temperature of the system was allowed to fluctuate around a mean of 310 K by employing Langevin dynamics^50^ with a collision frequency of 2/ps. Standard simulation protocols were used for the solvated protein cases^51^.

For the protein-lipid multicomponent system, the following simulation protocols were used. We followed rigorous energy minimization steps (3) to “relax” the system by eliminating any energetically unfavourable interactions that might have occurred while preparing the system using CHARMM-GUI. Minimisation was done in three steps: (1) minimised the solvent molecules holding the lipids and protein fixed by using position restraints with a force constant of 250 kcal/mol/Å^2^, (2) minimised the solvent and the lipid keeping the protein fixed and (3) finally all the atoms in the systems were set free of restraints and allowed to relax. We ran 5000 cycles of minimization steps in all three steps. The temperature of the energy minimised system was increased to 310 K linearly in two steps (0–100 K, 100–310 K). To avoid excessive solute fluctuations, we applied a weak restraint (10 kcal/mol/Å^2^) on the protein and lipid residues. Langevin dynamics with a collision frequency of 1.0/ps was applied for temperature equilibration at constant volume. Each of the equilibration steps involved 100 ps of MD runs in NVT ensembles. After the heating steps, the weak restraints on the protein and lipid residues were released in steps by carrying out four MD simulations, each of 500 ps, in NPT ensembles. Monte Carlo barostat (barostat = 2) with pure semi-isotropic (ntp = 3) pressure scaling was used to achieve a constant pressure of 1 atm. To reduce buckling of the bi-layer, a constant surface tension (0.0 dyne/cm) was applied at the membrane-liquid (csurften = 3) interface^52^. Finally, we ran MD “production run” for 1000 ns with an integration time step of 2 ps in NPT ensemble.

### Software used for analysis

Software used for data analysis and presentations are Origin, PyMol, UCSF chimera and Adobe photoshop.

## Supplementary Information

Supplementary Information.

## Data Availability

The pdb coordinates generated and/or analysed during the current study are not publicly available because these are generated through homology modelling. But these coordinate files will be available from the corresponding author on reasonable request. An important result of molecular dynamics simulation has been provided in the form of a movie as supplementary material. Other MD simulation results presented here will also be available in the form of movie on reasonable request.

## References

[1] Meneghetti F (2016). Iron acquisition pathways as targets for antitubercular drugs. Curr. Med. Chem..

[2] Palmer LD, Skaar EP (2016). Transition metals and virulence in bacteria. Annu. Rev. Genet..

[3] Sheldon JR, Laakso HA, Heinrichs DE (2016). Iron acquisition strategies of bacterial pathogens. Microbiol. Spectr..

[4] Cornelissen CN (2018). Subversion of nutritional immunity by the pathogenic Neisseriae. Pathog. Dis..

[5] Raymond KN, Dertz EA, Kim SS (2003). Enterobactin: An archetype for microbial iron transport. Proc. Natl. Acad. Sci..

[6] Eakanunkul S (2005). Characterization of the periplasmic heme-binding protein ShuT from the heme uptake system of *Shigella dysenteriae*. Biochemistry.

[7] Hider RC, Kong X (2010). Chemistry and biology of siderophores. Nat. Prod. Rep..

[8] Choby JE, Skaar EP (2016). Heme synthesis and acquisition in bacterial pathogens. J. Mol. Biol..

[9] Starr J (2014). Siderophore receptor-mediated uptake of lactivicin analogues in gram-negative bacteria. J. Med. Chem..

[10] Celia H (2016). Structural insight into the role of the Ton complex in energy transduction. Nature.

[11] Hollenstein K, Dawson RJ, Locher KP (2007). Structure and mechanism of ABC transporter proteins. Curr. Opin. Struct. Biol..

[12] Borths EL, Poolman B, Hvorup RN, Locher KP, Rees DC (2005). In vitro functional characterization of BtuCD-F, the *Escherichia coli* ABC transporter for vitamin B 12 uptake. Biochemistry.

[13] Woo JS, Zeltina A, Goetz BA, Locher KP (2012). X-ray structure of the *Yersinia pestis* heme transporter HmuUV. Nat. Struct. Mol. Biol..

[14] Naoe Y (2016). Crystal structure of bacterial haem importer complex in the inward-facing conformation. Nat. Commun..

[15] Qasem-Abdullah H, Perach M, Livnat-Levanon N, Lewinson O (2017). ATP binding and hydrolysis disrupt the high-affinity interaction between the heme ABC transporter HmuUV and its cognate substrate-binding protein. J. Biol. Chem..

[16] Tamura K, Sugimoto H, Shiro Y, Sugita Y (2019). Chemo-mechanical coupling in the transport cycle of a heme ABC transporter. J. Phys. Chem. B.

[17] Tamura K, Sugita Y (2020). Free energy analysis of a conformational change of heme ABC transporter BhuUV-T. J. Phys. Chem. Lett..

[18] Wyckoff EE, Stoebner JA, Reed KE, Payne SM (1997). Cloning of a *Vibrio cholerae* vibriobactin gene cluster: Identification of genes required for early steps in siderophore biosynthesis. J. Bacteriol..

[19] Payne SM, Mey AR, Wyckoff EE (2016). Vibrio iron transport: Evolutionary adaptation to life in multiple environments. Microbiol. Mol. Biol. Rev..

[20] Occhino DA, Wyckoff EE, Henderson DP, Wrona TJ, Payne SM (1998). *Vibrio cholerae* iron transport: Haem transport genes are linked to one of two sets of tonB, exbB, exbD genes. Mol. Microbiol..

[21] Wyckoff EE, Schmitt M, Wilks A, Payne SM (2004). HutZ is required for efficient heme utilization in *Vibrio cholerae*. J. Bacteriol..

[22] Agarwal S, Dey S, Ghosh B, Biswas M, Dasgupta J (2017). Structure and dynamics of Type III periplasmic proteins VcFhuD and VcHutB reveal molecular basis of their distinctive ligand binding properties. Sci. Rep..

[23] Rice AJ, Park A, Pinkett HW (2014). Diversity in ABC transporters: Type I, II and III importers. Crit. Rev. Biochem. Mol. Biol..

[24] Dey S, Biswas M, Sen U, Dasgupta J (2015). Unique ATPase site architecture triggers cis-mediated synchronized ATP binding in heptameric AAA+-ATPase domain of flagellar regulatory protein FlrC. J. Biol. Chem..

[25] Buckstein MH, He J, Rubin H (2008). Characterization of nucleotide pools as a function of physiological state in *Escherichia coli*. J. Bacteriol..

[26] Korkhov VM, Mireku SA, Veprintsev DB, Locher KP (2014). Structure of AMP-PNP-bound BtuCD and mechanism of ATP-powered vitamin B12 transport by BtuCD-F. Nat. Struct. Mol. Biol..

[27] Chen CC, Hwang JK, Yang JM (2009). (PS)2–v2: Template-based protein structure prediction server. BMC Bioinform..

[28] Jumper J (2021). Highly accurate protein structure prediction with AlphaFold. Nature.

[29] Case DA (2018). AMBER 2018.

[30] Jo S, Kim T, Iyer VG, Im W (2008). CHARMM-GUI: A web-based graphical user interface for CHARMM. J. Comput. Chem..

[31] Weng J, Fan K, Wang W (2012). The conformational transition pathways of ATP-binding cassette transporter BtuCD revealed by targeted molecular dynamics simulation. PLoS ONE.

[32] Trott O, Olson AJ (2009). AutoDock Vina: Improving the speed and accuracy of docking with a new scoring function, efficient optimization, and multithreading. J. Comput. Chem..

[33] Chen J, Lu G, Lin J, Davidson AL, Quiocho FA (2003). A tweezers-like motion of the ATP-binding cassette dimer in an ABC transport cycle. Mol. Cell.

[34] Moradi M, Tajkhorshid E (2013). Mechanistic picture for conformational transition of a membrane transporter at atomic resolution. Proc. Natl. Acad. Sci..

[35] Mani RS, Karimi-Busheri F, Cass CE, Weinfeld M (2001). Physical properties of human polynucleotide kinase: Hydrodynamic and spectroscopic studies. Biochemistry.

[36] Gedeon PC, Thomas JR, Madura JD (2015). Accelerated molecular dynamics and protein conformational changes: A theoretical and practical guide using a membrane embedded model neurotransmitter transporter. Methods Mol. Biol..

[37] Moradi S, Nowroozi A, Shahlaei M (2019). Correction: Shedding light on the structural properties of lipid bilayers using molecular dynamics simulation: A review study. RSC Adv..

[38] Meagher KL, Redman LT, Carlson HA (2003). Development of polyphosphate parameters for use with the AMBER force field. J. Comput. Chem..

[39] Wang J, Wang W, Kollman PA, Case DA (2006). Automatic atom type and bond type perception in molecular mechanical calculations. J. Mol. Graph. Model..

[40] Li P, Merz KM (2016). MCPB.py: A python based metal center parameter builder. J. Chem. Inf. Model..

[41] Li, P. & Merz, K. M. *Building Bonded Model for a Heme Group with MCPB.py* (2015). http://ambermd.org/tutorials/advanced/tutorial20/mcpbpy_heme.htm (Accessed 8 August 2021).

[42] Jakalian A, Jack DB, Bayly CI (2002). Fast, efficient generation of high-quality atomic charges. AM1-BCC model: II. Parameterization and validation. J. Comput. Chem..

[43] Wang J, Wolf RM, Caldwell JW, Kollman PA, Case DA (2004). Development and testing of a general amber force field. J. Comput. Chem..

[44] Maier JA (2015). ff14SB: Improving the accuracy of protein side chain and backbone parameters from ff99SB. J. Chem. Theory Comput..

[45] Dickson CJ (2014). Lipid14: The Amber lipid force field. J. Chem. Theory Comput..

[46] Price DJ, Brooks CL (2004). A modified TIP3P water potential for simulation with Ewald summation. J. Chem. Phys..

[47] Kräutler V, van Gunsteren WF, Hünenberger PH (2001). A fast SHAKE algorithm to solve distance constraint equations for small molecules in molecular dynamics simulations. J. Comput. Chem..

[48] Berendsen HJC, Postma JPM, van Gunsteren WF, DiNola A, Haak JR (1984). Molecular dynamics with coupling to an external bath. J. Chem. Phys..

[49] Darden T, York D, Pedersen L (1993). Particle mesh Ewald: An N ⋅log(N) method for Ewald sums in large systems. J. Chem. Phys..

[50] Pastor RW, Brooks BR, Szabo A (1988). An analysis of the accuracy of Langevin and molecular dynamics algorithms. Mol. Phys..

[51] Roy R, Ghosh B, Kar P (2020). Investigating conformational dynamics of Lewis Y Oligosaccharides and elucidating blood group dependency of cholera using molecular dynamics. ACS Omega.

[52] Zhang Y, Feller SE, Brooks BR, Pastor RW (1995). Computer simulation of liquid/liquid interfaces. I. Theory and application to octane/water. J. Chem. Phys..

