# Supplementary material for: Mechanistic insights of ABC importer HutCD involved in heme internalization by Vibrio cholerae
Source: Sci Rep. 2022 May 3;12:7152. doi: 10.1038/s41598-022-11213-9 (PMC9065009; doi:10.1038/s41598-022-11213-9)
Supplement: Supplementary file 1 — Supplementary Information. [file 41598_2022_11213_MOESM1_ESM.docx]

**
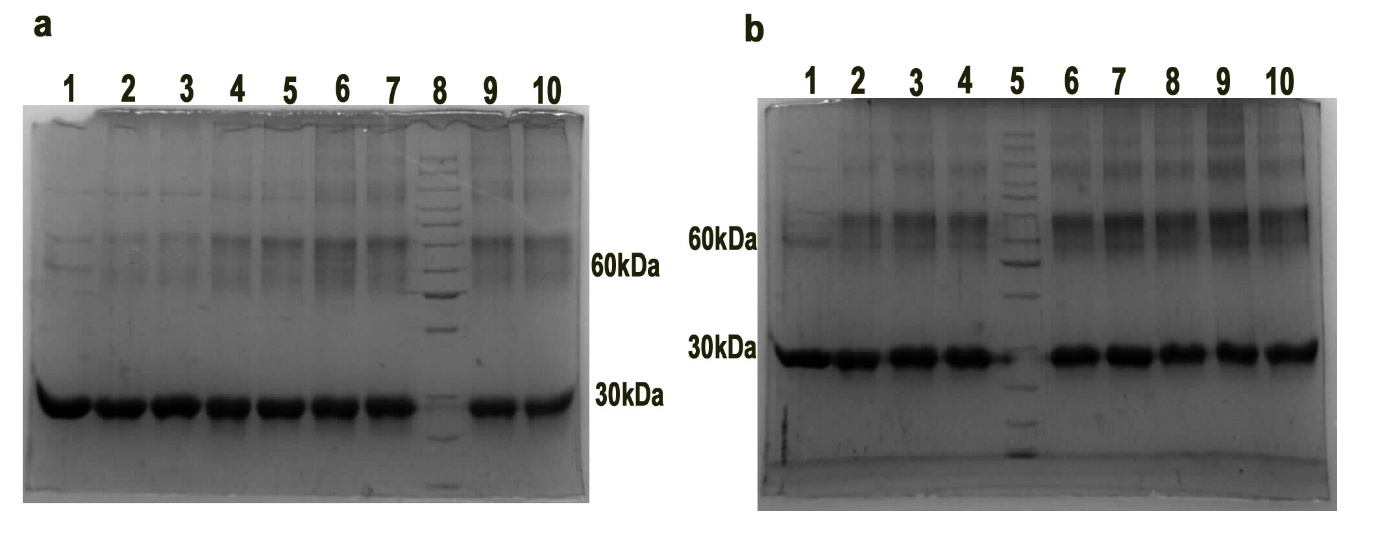
**

Fig. S1: Crosslinking of HutD with Glutaraldehyde (a) in Nt-free state and (b) after incubation AMP.PNP.  a: (L1: Control, L2-L7: after treatment with 0.005-0.04% of Glutaraldehyde, L8: MWM, L9-L10: after treatment with 0.045-0.05% of Glutaraldehyde). b: (L1: Control, L2-L4: after treatment with 0.005%, 0.01%, and 0.02% of Glutaraldehyde, L5: MWM, L6-L10: after treatment with 0.03-0.05% of Glutaraldehyde).

**Table S1: Mutation plans of probable crucial residues of HutD:**

| **HutD residue** | **Probable role** | **Mutation** |
| --- | --- | --- |
| **Arg18** | **Stabilization of sugar of ATP** | **R18A** |
| **Lys44** | **Residue from the WA motif, crucial for stabilizing Phosphate groups of ATP** | **K44A** |
| **Asp166** | **Part of Walker B** | **D166A** |
| **Glu167** | **Walker B residue, expected to bind Mg^2+^ for ATP hydrolysis** | **E167A** |


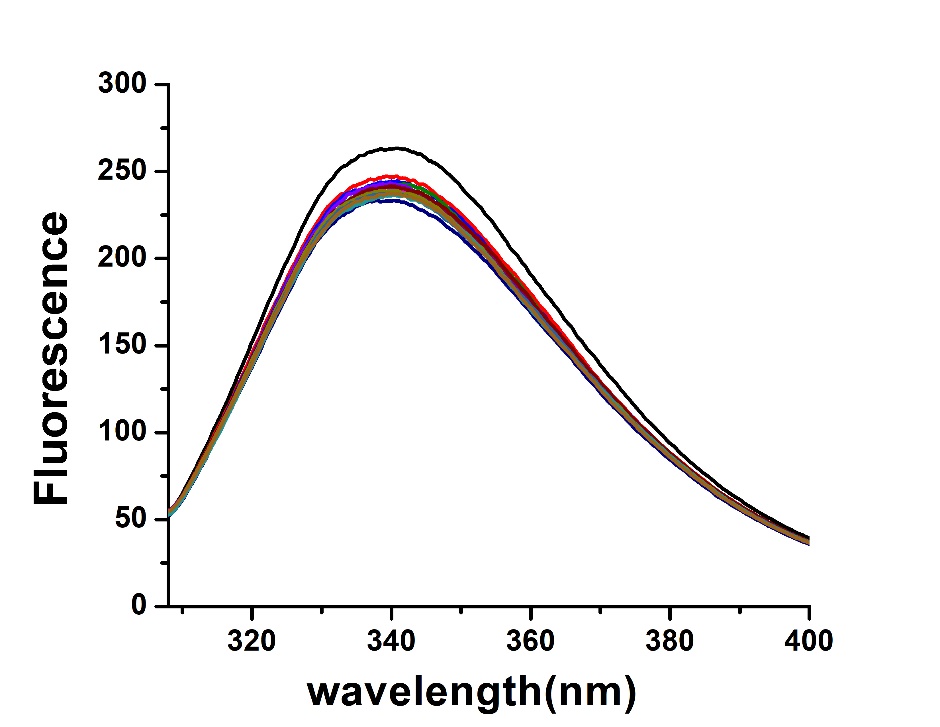


Fig S2: Negligible changes in fluorescence of Trp upon AMP.PNP binding to HutD were observed with excitation at wavelength of 295 nm and the emission spectra were recorded between 308 nm and 400 nm with slit widths of 5 nm for both excitation and emission.


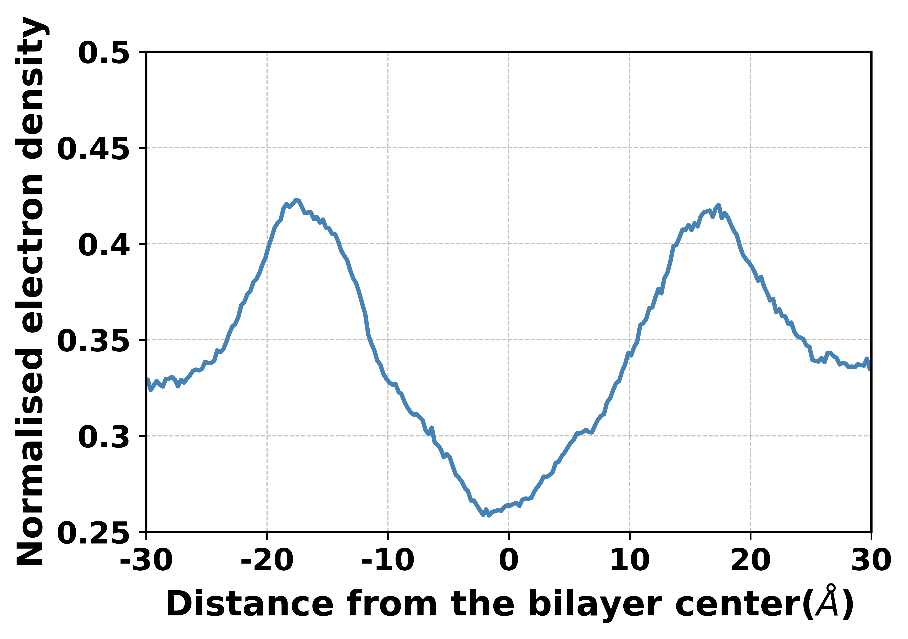


Fig. S3: Time averaged electron density profile of the DMPC bilayer extracted from the Heme bound simulation. Both the leaflets of the bilayer were intact and maintained the bilayer thickness during the course of dynamics.


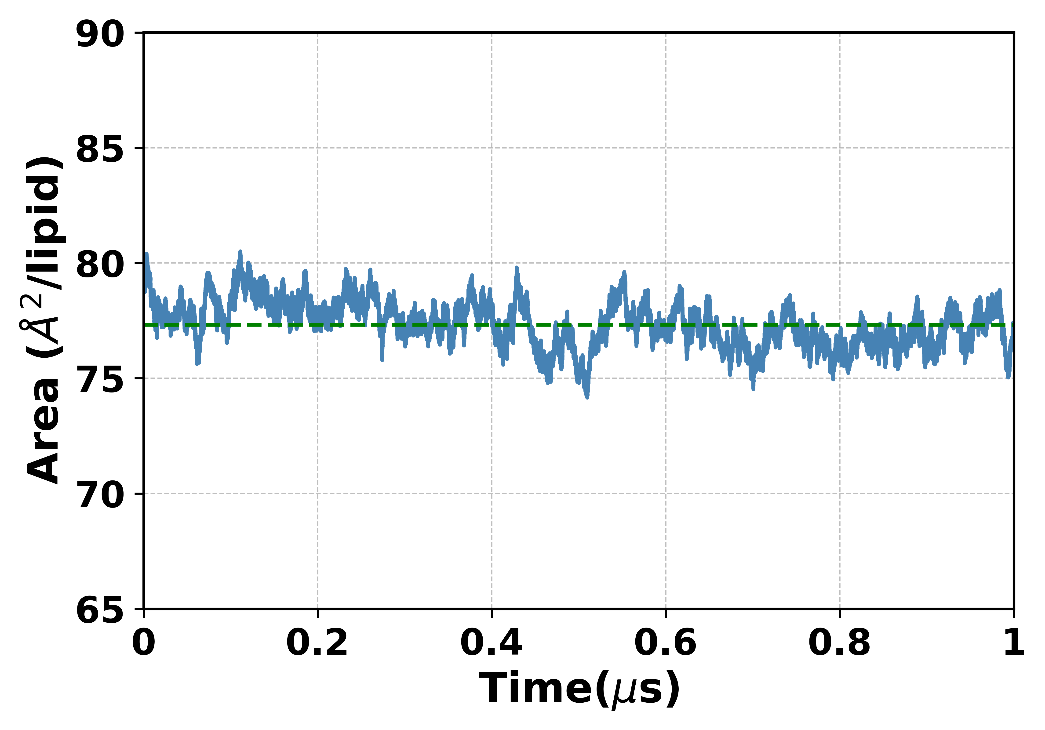


Fig. S4: The time evolution of the average area per lipid molecule is plotted. Fluctuations of the average lipid area about a mean value indicate convergence and stability of the bilayer during the course of simulation.

**Table S2: Heme interaction with HutD protomers in HutCD assembly (detailed with residue numbers and specific loops):**

| **Frames(ns)** | **Heme interaction with HutD2**  **Residue no.s and specific loop** | **Heme interaction with HutD1**  **Residue no.s and specific loop** |
| --- | --- | --- |
| 123 | [SLT(88-90), Q-loop]   - Also interacts with TM5 of A-chain T156 | No interaction |
| 172 | S87,S88,T90 OF Q LOOP | S88 OF QLOOP |
| 289 | S88, S138, SALD(170-174,D-loop) | No interaction |
| 414 | S88, S138, SALD(170-174,D-loop) | QQ(85-86, Q-loop),K49 |
| 1019 | Q85, S88 | S88 |
| 1063 | QQ(85-86, Q-loop), S88,E167, D170 | No interaction |
| 1227 | QQ(85-86, Q-loop), S88,E167, D170 | S88 |
| 1450 | N40(P-loop), QQ(85,86), S88, Q143(ABC-signature motif),E167(walker),S170(D-loop) | No interaction |
| 1793 | Q85, SA(170-171),E167 | Q86 S88 |
| 1903 | GAGK(41-44),K49,T46(all from P-loop) Q85 | No interaction |
| 2058 | No interaction with C and D chain, It interacts with TM helices K244(A-chain), F343(A-chain), Glu575(B-chain) | R18 GPN(40-42) C-chain |
| 2852 | K49, E167, SA(170-171), Q85 | No interaction |
| 3618 | S17 | No interaction |
| 4614 | E167, SA(170-171), Q85 | No interaction |
| 5113 | S88, E167, S170, Q85 | No interaction |
| 6111 | QQS(85-87), SA(170-171) ,G41, E167 | T90(Q-loop) |
| 7608 | LPQ(83-85), S87, DEPTSAL(166~~170~~-172),Q143 | No interaction |
| 9603 | Q85,S88,L89,T90 | QSS(86-88) |
| 12597 | QSSLT(86-90) | No interaction |
| 14593 | No interaction | Q86, SLT(88-90) L-loop residues LGAE of TMD-A |
| 15092 | no | S88   - HutC2-I342 |
| 16090 | no | - HutC2-I342, HutC1-T156 |
| 26568 | Q85 | No interaction |
| 28564 | Q86 | No interaction |
| 34552 | T90 | No interaction |
| 38544 | Q85, S87 | No interaction |
| 40539 | S87,S88,T90(Qloop) | No interaction |
| 40884 | S88,T90 | No interaction |
| 41122 | Q86,S88 | No interaction |
| 43034 | QSS(86-88) | No interaction |
| 44032 | SSLT(Q-loop) | No interaction |
| 44531 | QS(86-87) | No interaction |
| 46883 | Q19,AGK(42-44), T46, QGK(217-219) | PSLS(135-138) |
| 47026 | GAGK(41-44,P-loop) | S132,LS(137-138),E141 |
| 47143 | No interaction | S132, SLSG(136-139) |
| 47160 | No interaction | S132,S135,S138 |
| 47162 | NGA(40-42,P-loop) | S135,S138,G139 |
| 47168 | K44 | S138(ABC signature motif) |
| 47174 | No interaction | S136 |
| 47205 | No interaction | S132,S135,L136  HEME IS MOVING OUT |
| 47250 | No interaction | S132 |

*Criteria: Selected all atoms/bonds of any residue that meet the criteria ≤ 4Å from heme.*
